# Multimodal pulse oximeters to support the integrated management of childhood illnesses: A usability and diagnostic accuracy assessment from a multi-country hybrid type 2 study

**DOI:** 10.1371/journal.pgph.0004655

**Published:** 2026-03-26

**Authors:** Helen L. Storey, Tessa L. Fielding, Julia Mwesigwa, Rebecca K. Green, Megan E. Parker, Anmol Jacob, Samwel Lwambura, Mumbe Kitonga, Leila Maina, Mansi Tyagi, Alice Mwikamba, Caroline Ngunu, Anuj Kumar Pandey, Ndèye Marème Sougou, Jean Tine, Angharad Steele, Tedila Habte, Misganu Endriyas, Kevin Baker, Viviana Rivas, Sayali Walke, Maymouna Ba, Andolo Miheso, Deusdedit Mjungu, Kovid Sharma, Mira Emmanuel-Fabula, Mike Ruffo, Ambrose Agweyu, Shally Awasthi, S. N. Singh, Divas Kumar, Grace Mhalu, Papa Moctar Faye, Ousmane Ndiaye

**Affiliations:** 1 PATH, Seattle, Washington, United States of America; 2 PATH, Kampala, Uganda; 3 King George Medical University, Lucknow, India; 4 Ifakara Health Institute, Dar Es Salaam, Tanzania; 5 Kenya Pediatric Research Consortium, Nairobi, Kenya; 6 Université Cheikh Anta Diop, Dakar, Senegal; 7 Malaria Consortium, London, United Kingdom; 8 Malaria Consortium, Addis Ababa, Ethiopia; 9 PATH, Minneapolis, Minnesota, United States of America; 10 PATH, Dakar, Senegal; 11 PATH, Nairobi, Kenya; 12 PATH, Dar Es Salaam, Tanzania; 13 PATH, Lucknow, India; 14 PATH, Geneva, Switzerland; 15 KEMRI-Wellcome Trust Research Programme, Kenya; 16 London School of Hygiene and Tropical Medicine, United Kingdom; PLOS: Public Library of Science, UNITED STATES OF AMERICA

## Abstract

Nearly 5 million children die each year of preventable causes, with pneumonia being a key contributor. The Integrated Management of Childhood Illnesses guidelines improve health care workers’ diagnostic and management capabilities by relying mostly on clinical signs. Though there have been successes, challenges in the consistent application of IMCI and the accurate diagnosis of conditions like hypoxemia remain. Next generation pulse oximeters add functionality to stand alone pulse oximeters, like measurement of respiratory rate, temperature, and hemoglobin. While the TIMCI project sought to address gaps in the introduction of pulse oximetry in India, Kenya, Senegal, and Tanzania, research was also conducted to strengthen the market for multimodal pulse oximetry (PO) devices by filling evidence gaps around ideal product attributes and the validation of available and near to market photoplethysmography-derived clinical measurement tools (medical device and smartphone-based). A mixed-methods evaluation measured usability and diagnostic accuracy using multimodal PO devices among primary care providers in the four countries. Results showed good usability, minimal user errors, and high satisfaction and system usability scores across all devices. Additionally, across all age categories, device performance for hypoxemia, tachycardia, and fever exceeded 80% agreement; respiratory rate measurements exhibited greater variability in percent agreement between devices. A target product profile and an open-source data repository were developed to further advance device development and market alignment. This research provided data on the performance of various multimodal PO devices, considering different form factors and product attributes. Technological progress continues to expand opportunities for the collection of clinical measurements and data. Supporting providers with decision support and automated documentation tools ensures that the information generated is actionable and utilized, while an emphasis on integrated technologies is essential to maximize provider capabilities and improve the diagnosis and management of childhood illnesses in low- and middle-income countries.

## Introduction

The Integrated Management of Childhood Illnesses (IMCI) strategy, introduced in the mid-1990s, aims to reduce child mortality and morbidity by providing integrated guidelines to help health care workers (HCWs) effectively diagnose and manage the major causes of childhood illness, through a structured approach to case management [1–3]. In 2023, an estimated 4.8 million children under five died of preventable causes, mostly due to pneumonia (20%), malaria (16%), and diarrhea (15%) [4]. Among these deaths, fever is a common symptom often overlapping with coughing and fast breathing. Despite progress in malaria detection and treatment, diagnosing and managing children with other diseases remains challenging. Identifying risk factors like hypoxemia and other conditions of severe illness is essential for guiding treatment decisions to avert death.

Challenges exist with the use of IMCI such as inconsistent adherence by health workers [4–8], and sole reliance on clinical signs to support identification and treatment of severely sick children [9–13], which is inadequate for detecting hypoxemia [14,15]. Hypoxemia, or low blood oxygen saturation, can be easily measured with a pulse oximeter (PO). Use of PO in hospitals and primary care settings saves lives and resources [13,16,17], and its global use and awareness has grown since the Covid-19 pandemic [18]. Respiratory rate is essential for diagnosing pneumonia in IMCI, but it is often under-measured and subject to human error, particularly in young children with fast breathing [8,19–21]. Integrated pulse oximeters that measure blood oxygen saturation and respiratory rate are now commercially available, and though challenges with manually and automatically measuring respiratory rate remain [21], early studies have demonstrated positive usability and acceptance of this multimodal PO technology among community health workers [21,22], as well as good performance in hospital settings [23,24]. Nevertheless, more evidence is needed to build confidence in the capabilities of these devices in primary health care (PHC) settings and demonstrate the operational feasibility of their integration into existing IMCI care practices, especially with the added functionality of temperature measurement.

Furthermore, medical device manufacturers are leveraging photoplethysmography (PPG), the underlying technology in pulse oximeters and a measurement of the circulatory system, to detect other health indicators using machine-learning/artificial intelligence (ML/AI) models. Based on light absorption or reflection, changes in blood flow are detected as changes in light intensity through the blood and tissues [24], allowing additional measurements such as heart rate, respiration, blood pressure, and anemia. Consumer products such as smartwatches and smartphone applications also detect light reflective PPG measurement using integrated sensors such as the camera and flashlight. As smartwatch and smartphone-based sensors become more powerful [25], it is important to consider the potential role of PPG derived smartphone-based clinical screening tools to digitize health data, facilitate risk-based stratification of patients, and support faster decision-making on individual and population-based care. While consumer products are not replacements for medical devices, the current methods in IMCI for assessing fast breathing by counting and anemia by consideration of pallor [10–13], are subjective and result in considerable variation across providers. Even still, these assessments are critical for frontline health workers to inform decision-making [26], and highlight the need for clinical screening tools to expand the reach of medical devices, if they can be reliable and user friendly. Demonstrating that new tools perform as intended in the target context of use builds the evidence needed for introduction and eventual scale-up.

A key barrier that manufacturers face in advancing next generation multimodal POs is validating new devices or algorithms, particularly among hard to reach populations like children, and not having clear guidance on necessary product attributes. Developing new medical devices requires significant investment, and when validation is expensive, demand is uncertain, and the market is price sensitive, companies may view the risk as too high, despite the potential to save lives. Product development partners derisk research and development for new products where the potential for health impact is greater than the perceived return on investment for a company. Sustaining a low margin, or no margin public health product may be more feasible for a company if the upfront investment is minimized.

The Tools for the Integrated Management of Childhood Illness (TIMCI) project was a 5-year Unitaid-funded project focused on the interventions of pulse oximetry and clinical decision support algorithms (CDSAs) in primary care. Results from research on the introduction and evaluation of PO and CDSAs in primary care in Kenya, Senegal, Tanzania, and Uttar Pradesh, India are reported elsewhere [27,28]. To strengthen the market for new multimodal PO devices, research was also conducted to fill evidence gaps around ideal product attributes, and the validation and operational fit of available and near to market PPG-derived clinical measurement tools (medical device and smartphone-based screening technologies) through a hybrid type 2 study design. The mixed methods type 2 effectiveness-implementation study measured the performance and feasibility of identified multimodal pulse oximeter devices by primary care providers in Kenya, Senegal, Tanzania, and Uttar Pradesh, India. Research was also conducted to define the requirements of next generation multimodal PO devices through a target product profile (TPP) that communicates optimal and minimum product attributes, and an open-source data repository of reference measurements from the diagnostic accuracy study that aims to catalyze PPG-derived, multimodal PO medical device and smartphone-based clinical screening technology development. Here we report the performance results of the hybrid type 2 study with a manufacturer-agnostic perspective and share resources from the TPP development and the open-source data repository.

## Methods

### Ethics statement

Before evaluation by ethical review committees, the research described in this manuscript underwent scientific merit review by two separate reviewers. The study was then reviewed independently by ethical review boards in each country. For Tanzania, this included the Ifakara Health Institute IRB and the National Health Research Ethics Committee. For India, this included a) the National Health Ministry, Uttar Pradesh, b) the Institutional Ethics Committee of King George Medical University and c) the Health Ministry’s Screening Committee of the Indian Council of Medical Research. For Senegal, this was the Comité National d’Ethique por la Recherche en Santé. And for Kenya this was the Amref Health Africa Ethics and Scientific Review Committee and the National Council of Science and Technology. Written informed consent was obtained from all providers and caregivers of the children involved in this study.

**Inclusivity in research:** Additional information regarding the ethical, cultural, and scientific considerations specific to inclusivity in global research is included in supplemental information (S2 Checklist).

### TIMCI and parent study

The TIMCI project aimed to support healthcare providers to identify and manage severe illness, by introducing pulse oximetry and CDSAs to primary care facilities in India, Kenya, Senegal and Tanzania. To address evidence gaps, an evaluation was designed to include pragmatic cluster randomized controlled trials in India and Tanzania (NCT04910750), and quasi-experimental pre-post studies in Kenya and Senegal (NCT05065320), complemented by embedded mixed-method studies in all countries [29]. TIMCI also aimed to accelerate the development and market entry of non-invasive devices that augment the measurement features of standard pulse oximeters. These devices, called multimodal pulse oximeters, measure additional vital signs such as respiratory rate, temperature, and/or hemoglobin, that could improve healthcare providers’ ability to accurately diagnose, treat, and/or refer their patients. Barriers to market entry of multimodal pulse oximeters for use in LMICs have included lack of information to inform appropriate product design, as well as uncertain demand. To address gaps, a TPP was developed, and a field validation was conducted to evaluate the accuracy and operational feasibility of select multimodal pulse oximeters either in or near to market.

### TPP development

The TPP was developed through a multi-phase process, starting with a literature review and expert interviews to develop a first draft. Workshops were then conducted in Kenya, Tanzania, and Senegal to obtain feedback on the draft TPP from a national stakeholder perspective. The workshops took place either in person (Senegal, Tanzania) or virtually (Kenya) and included interactive, human-centered design activities led in small groups or individually. Participants were selected for their expertise in child health research, primary care practice, and national IMCI guideline development and implementation. Following the workshops, an online survey was conducted to assess agreement on the minimum and optimal requirements for 23 product attributes. For each requirement, respondents were asked to indicate whether they “agree”, “mostly agree”, “neither agree or disagree”, “mostly disagree”, “fully disagree”, or “other (do not have expertise to comment)”. A predefined agreement threshold of at least 60% selecting “agree”, “mostly agree”, or “neither agree or disagree” was set in advance. Finally, select manufacturers (n = 7) were engaged in a discussion where the draft TPP was shared, and challenges and opportunities with key product attributes were explored. Further details on development are available in supplemental information (S1 Text) and the final TPP is accessible at https://www.path.org/our-impact/resources/multimodal-pulse-oximeter-tpp.

### Type 2 hybrid study

#### Study design.

**Fig 1:**
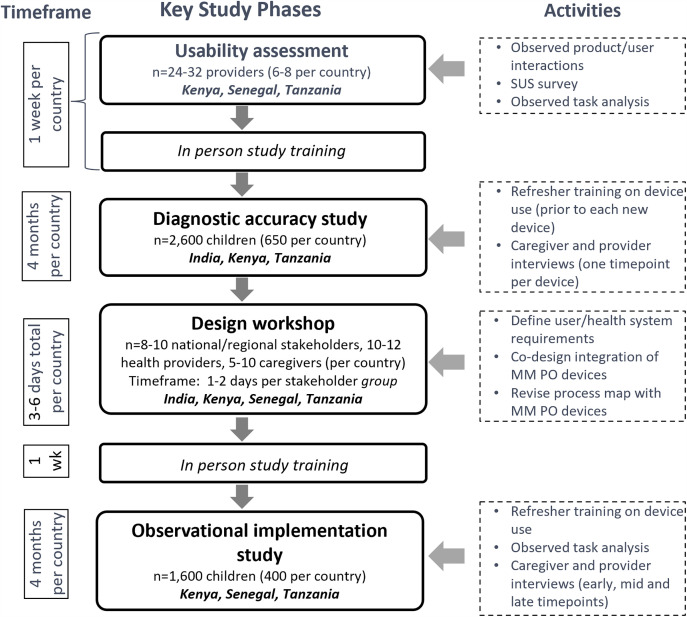
Type 2 hybrid study flow chart by key phases, activities, and timeframe.

The objective of this study was to evaluate identified multimodal pulse oximeter devices used by primary care providers in Kenya, Senegal, Tanzania, and Uttar Pradesh, India. To assess the performance and feasibility of multimodal PO devices a hybrid design was used to conduct a prospective mixed methods diagnostic accuracy and implementation study in two primary care facilities in each country [27,28]. (Fig 1) Recruitment occurred from 15 May 2023–18 December 2023 in Tanzania, 28 August 2023–27 December 2023 in India, 24 May 2023–09 January 2024 in Senegal, and 16 March 2023–15 December 2023 in Kenya. In this paper we discuss the results from the usability and diagnostic accuracy study.

#### Usability assessment.

**Fig 2:**
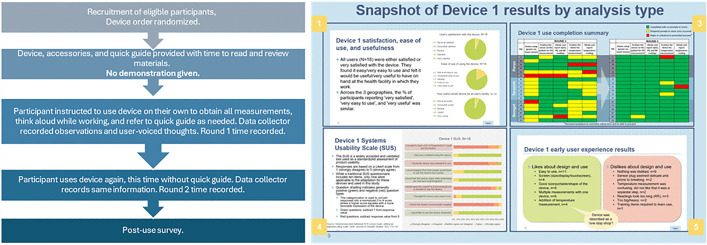
Workflow diagram for usability assessment and example results for each device.

To measure the usability and acceptability of the devices by healthcare providers and caregivers at the primary care level, a usability assessment was conducted. The usability assessment observed user-product interactions over 2 rounds of use to measure error modes and rates and time to result. A post-use survey measured user satisfaction among primary care providers. These assessments took place in January and February of 2023. All multimodal PO devices were assessed by 6–8 healthcare providers per country, which identifies around 80% of common user errors [30,31]. Each provider had familiarity with general POs in day-to-day practice and assessed all devices. Device order was randomized for each provider. Detailed observations were recorded using structured data collection forms and a systems usability score was used to assess usability across devices [32,33] (Fig 2).

**Fig 3:**
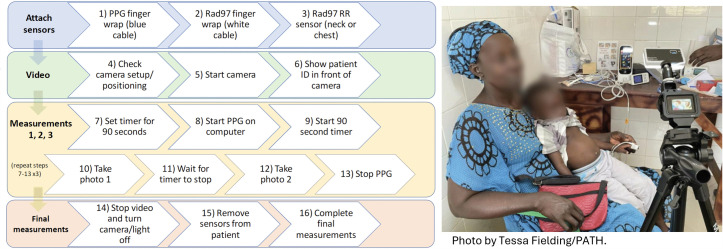
Workflow diagram used by research assistants during diagnostic accuracy study data collection, and illustration of positioning of participants and devices.

**Diagnostic accuracy study:** The primary objective of the diagnostic accuracy study was to determine the performance of available multimodal PO devices compared to an accepted reference standard, by primary care providers assessing children 0–59 months seeking care at the primary care level. A cross-sectional diagnostic accuracy study compared the index devices to an accepted reference standard to measure accuracy. The research activity occurred only after the patient received routine care according to current practices in the facility. A convenience approach was used for recruitment and for each participant, one index device and all reference devices were used simultaneously in 3 identical measurements recorded in sequence. Video recordings were captured as a single file, which were redacted of identifying attributes and edited to smaller clips for annotation purposes. Calmness and perfusion index were noted, as well as any device errors or issues preventing complete data collection for each participant. Measurements obtained at the same time from index and reference devices were compared for agreement. (Fig 3) The following indicators of performance were assessed: Bland Altman plots, intraclass correlation coefficients, confusion matrices for percent agreement, and mean absolute error. Following device assessment, semi-structured interviews were performed with primary care providers and caregivers to assess acceptability of the index devices. For each device assessed, 5–10 caregivers were surveyed for their perceptions on acceptability of each device. Device specific results are presented without attribution to product name. All products have strengths and weaknesses and continue to be improved through ongoing manufacturer updates. Product evaluations are specific to the product version at the time of the study.

#### Study population.

The study took place in four countries: Kenya, Senegal, Tanzania and India (Uttar Pradesh). Two facilities per country were selected in partnership with research partners and country stakeholders. Adjusting for study timeline constraints, the usability assessment was conducted in Kenya, Tanzania, and Senegal, while the diagnostic accuracy study was conducted in Kenya, Tanzania, and India. Index and reference measurements were collected on children 0–59 months seeking care at the primary care facility. Due to varying device fit and performance based on size, three age categories were evaluated and defined as: under 2 months (0–1), 2 months to under 12 months (2–11), and over 12 months to under 60 months (12–59). For devices with prior evidence of use in children, all three age categories were assessed, while devices without prior use data in children were only assessed in the 12–59 months age category. Evaluating older children first provided a chance to demonstrate feasibility with those who were less likely to encounter issues related to smaller fingers or increased movement. All facilities had experience with pulse oximetry use. The study enrolled children 0–59 months presenting with an illness, and for whom caregivers provided written consent. Children were excluded from the study if they were in the immediate post-natal period or first day of life, presenting for care due to trauma, admitted for inpatient care, were critically ill requiring emergency or immediate referral and care, or caregivers did not provide written consent.

#### Device measurements.

**Index devices:** A landscaping of PO and multimodal PO device manufacturers was conducted to identify devices for evaluation. A combination of desk research, and outreach to relevant partners, stakeholders and networks identified 16 potential technologies. Following discussions with manufacturers to align products to the TPP, and in-house verification benchmarking in the PATH engineering lab in Seattle, 5 technologies were selected for inclusion in the hybrid study. Device 1 measures blood oxygen saturation, respiratory rate, pulse rate, and temperature, is a handheld instrument with a finger clip attached by cord and displays measurements on the device. Device 2 measures blood oxygen saturation, respiratory rate, and pulse rate, is a handheld instrument with a finger clip attached by cord and displays measurements on the device. Device 3 measures blood oxygen saturation, respiratory rate, and pulse rate, is a fingertip clip instrument, and displays measurements on the device. Device 4 measures blood oxygen saturation, respiratory rate, pulse rate, temperature, and additional clinical parameters that were not assessed in this study, is an instrument that the patient holds in the palm of their hand and connects to a tablet by Bluetooth to display measurements. And device 5 measures blood oxygen saturation, respiratory rate, pulse rate, and temperature, is a forehead band instrument, and connects to a tablet by Bluetooth to display measurements. From a regulatory perspective, device 1 is FDA approved and CE marked, device 5 is CE marked, and devices 2, 3, 4, and 6 are in development. (See S2 Text for detailed specifications of multimodal PO devices by product attribute)

Through our landscaping work on next generation PO devices and noninvasive anemia measurement [34], PPG-derived clinical measurement capabilities were also identified on smartphone platforms. Using existing sensors in the phone, some clinical measurements under development include pulse rate, blood oxygen saturation, respiratory rate, and anemia [21,24,35,36]. To assess the usability of this type of smartphone device, a consumer application available for download was used as a comparable form factor and user interface for measuring heart rate and respiratory rate, and results are presented as device 6. Additionally, an android device (Samsung Galaxy A13) was included in the performance study to noninvasively capture clinical data using the existing sensors, which included video data from placing a finger over the camera to derive PPG waveform, photos of eye conjunctiva, and photos of nailbeds. Children ages 2–11 months and 12–59 months were assessed, excluding the youngest age category due to the limited research on young babies and an abundance of caution to protect this vulnerable population. Because research to develop an algorithm that classifies the data based on a threshold is in development, this smartphone-derived clinical data is included in our data repository to support future research by others.

**Reference devices:** A reference standard for each clinical measurement was used at the same time as the index device to determine agreement of the devices. Abnormal clinical measurements are defined as follows for each clinical parameter based on reference measurement: blood oxygen saturation (<90%), pulse rate (>179 bpm for less than 12 months, > 139 bpm for 12–60 months), temperature (≥37.5C), and respiratory rate (>49 bpm for less than 12 months, > 39 bpm for 12–35 months, and >29 bpm for 36–60 months). (Table 1)

**Table 1:** Summary of primary and secondary reference measurements and devices used.

	Clinical measurement	Reference device	Purpose
**Primary**	**Blood oxygen saturation/ pulse rate**	Rad-97 with RD SET Rainbow Sensor (finger wrap) (https://www.masimo.com/products/continuous/rad97/, Masimo, USA),	Reference standard for blood oxygen saturation and pulse rate measurements.
	**Respiratory rate**	GoPro video of chest movement (https://gopro.com/en/ph/shop/cameras, GoPro, USA)	Videos were deidentified, trimmed, and annotated by a panel of experts and used as the reference standard for respiratory rate measurement. See methods in below respiratory rate annotation section.
	**Temperature**	TIE-240 non-contact infrared digital thermometer (HoMedics, USA)	Reference standard for temperature measurement. Used at the child’s forehead.
**Secondary**	**Respiratory rate**	Rad-97 acoustic respiratory rate	A third reference point for respiratory rate measurement. Not used as primary clinical value comparison.
	**Hemoglobin**	HemoCue 201 + Hb system (https://www.hemocue.us/hb-201/, HemoCue, Sweden)	Generating data for future product development.
	**Skin tone**	CR-20 Chroma meter device, (https://sensing.konicaminolta.us/us/products/cr-20-color-reader/, Konica Minolta, Japan) and Monk Skin Tone Scale reference sheets (https://skintone.google/)	Quantitative and qualitative measurement of skin tone due to the potential source of performance variability in pulse oximeters [37,38] Measured at a clean, clear area on the back of the hand.
	**PPG**	Custom raw PPG waveform device, developed by research partners at UCSF with the ADI MAX86171 optical front-end chip, 19-bit resolution and over 108 dB SNR, 2 LED transmissive sensors (660nm, and 910nm), a fixed LED drive and a wrapping fingertip probe (donated by Nihon Kohden, Japan) [39].	Generating data for future product development.
	**Anthropometry**	Scale, height board, tape measure	Height, weight, MUAC, and head circumference were recorded for analysis.

**Respiratory rate annotation:** Respiratory rate technologies utilize different methodologies such as accelerometers to measure chest movement, capnography to measure respiration, acoustics to measure breath sounds, and PPG to measure blood circulation in tissue [21]. In a controlled setting, capnography is the gold standard for respiratory rate measurement, however, a human counter is the current practice in primary care settings, and the preferred comparison for new technologies. To minimize interrater and intrarater variability in respiratory rate measurement by health care providers [9,40,19,39,37], videos were used to standardize the human counter, which has been used in previous studies [20,38]. Recording of the videos was exactly aligned to the time period of the index test measurements.

Prior to annotation, video recordings were edited into 60-second clips, with a maximum of three measurement clips per participant, according to the developed standard operating procedure. Each 60-second clip was reviewed and categorized to identify the highest quality measurement clip to use in annotation for each participant, resulting in a maximum of one measurement per participant.

To conduct the video annotation a panel of six reviewers was formed. Three clinical nurses and three health officers were recruited in Hawassa, Ethiopia. All reviewers had undergone previous training in line with the Integrated Management of Neonatal and Child Illnesses (IMNCI) strategy [41], and had practical experience managing sick children under five years of age. To ensure annotation in accordance with guidelines, and to standardize annotation between reviewers, the panel underwent a three-day training. The panel training was conducted in line with the standard operating procedure developed for the annotation, and included modules on respiratory rate counting, data collection methods and procedures, as well as instructions on how to use the annotation software tool (Philips Foundation, Belgium). This adapted software was used during training and data collection, using videos collected from a previous study [37]. The training was concluded with individual blinded competency tests where each reviewer was assigned five videos with known respiratory rates and reviewer accuracy was determined by a respiratory rate within ±2 bpm of the video’s known value. All reviewers met or exceeded the pass mark of 80% before commencing data collection [42].

For annotation, each reviewer was randomly assigned to one of three annotation groups each day (two reviewers per group). Videos were also assigned randomly and in proportion to the quality category designated to ensure videos of varying quality were distributed across the annotation groups. Breaths were marked using the annotation tool at the point of full chest expansion, with a full breath cycle being defined as the observation of one full inhalation and one full exhalation. Each video was annotated by both group members, and agreement was defined as a discrepancy in respiratory rate of ±2.5 breaths or less. If there was agreement, the annotation of the video was considered complete. If there was no agreement, the video was randomly assigned to a third panel member who was blinded to the results of the first two annotations. In addition to annotation and respiratory rate data collected through the annotation tool, supplementary data including annotator feedback was recorded. All data were recorded in SurveyCTO (Dobility, Inc, USA) before being exported for analysis.

#### Data management and analysis.

Research data was collected through structured data collection forms, surveys, case report forms, and video recording using REDCap (made available through grant UL1 TR002319) and reviewed daily for completeness and accuracy. No patient identifying information was collected as the focus of the evaluation was the provider and the device.

Usability was assessed by likert scale questions on ease of use and user confidence, error rates were classified by minor and critical errors, hands-on time to result refers to how long the device takes to provide measurements for all the clinical parameters it can evaluate and was measured by provider, and system usability scale (SUS) composite scores were calculated as described elsewhere [32]. To assess acceptability of the index devices by providers, a survey was administered containing open ended and Likert scale questions, as well as a semi-quantitative survey on product attributes. The diagnostic accuracy study was analyzed by clinical measurement (blood oxygen saturation, pulse rate, respiratory rate, or temperature) for each device and age category using Bland Altman plots, mean absolute error, and percent agreement, and following STARD guidelines [43,44]. The completed STARD checklist is available in supplemental information (S1 Checklist).

Baseline characteristics of participants, including providers and child/caregiver pairs, and usability assessment data were summarized using descriptive statistics. Bland Altman plots were generated for each clinical measurement, device and age category, to evaluate bias in the comparison of the index device to the reference device. Mean absolute error and standard deviations were calculated to give bias and upper/lower limits of agreement, describing the relative magnitude of measurement difference and giving insight into accuracy and precision with a single value. Directional drift indicated whether the index device measured high or low with respect to the reference value. Within each clinical measurement, units of measurement are the same so values are comparable. Bias and error were also evaluated after removing outlier values that were more than 3 standard deviations from the mean, indicating a likely erroneous value. For all the reference devices except annotated respiratory rate, 3 measurements were attempted for each child consultation, all conducted by a single provider. Measurement attempts were averaged together to give an overall percent agreement per participant and an overall percent agreement per age category. Four age categories were used for fast breathing in alignment with clinical cutoffs.

Using the method by Lu et. al. (2016) [45], the calculated sample size for the diagnostic accuracy study was 50 observations per targeted age category, rounding up to account for attrition or failed measurements. As an accuracy of at least ±2 breaths per minute translates to a maximum allowable difference of 2 plus the confidence interval of the limit of agreement, it was calculated that a mean difference of ≤1.3 with a standard deviation of 1.0 would have reasonable precision to detect a maximum allowable difference of 4.0 with a sample size of 47. Based on prior evidence of use in children, 3 devices were evaluated in all 3 age categories, 1 device was evaluated in the 2 older age categories, and 2 devices were evaluated only in the oldest age category. In total, enrollment of 650 children was the target of the diagnostic accuracy study per country.

All analyses were conducted by clinical measurement, device and age category. Quantitative analyses were performed in R using the BlandAltmanLeh and irr packages and conducted collaboratively using GitHub [46,47]. Nutrition measures were calculated using the zscorer package [48].

### Data repository

With approval from all reviewing ethics committees, reference measurements from the diagnostic accuracy study are available in a data repository to facilitate secondary research on noninvasive and smartphone-based technologies for children. The repository contains deidentified reference data collected from the patient monitor, thermometer, Hemocue device, chromameter, custom PPG device, anthropometric assessments, redacted evaluation videos, and associated data from the smartphone application. The repository is structured to meet ethical, legal, and scientific oversight requirements to allow secondary research for potential commercial use, similar to biorepositories developed previously [49]. The data repository governance plan was reviewed by all research partners and the PATH Office of Research Affairs. Database storage is maintained on an AWS platform and requests for access to the data can be made online [50].

## Results

To summarize form factors, Device 1 is a handheld unit with a corded finger clip that measures blood oxygen saturation, respiratory rate, pulse, and temperature, displaying results directly. Devices 2 and 3 also measure blood oxygen saturation, respiratory rate, and pulse; Device 2 is handheld with a corded clip, while Device 3 is a fingertip clip, both showing data on the device. Device 4, held in the palm, measures blood oxygen saturation, respiratory rate, pulse, and temperature, sending readings to a tablet. Device 5 is a forehead band measuring blood oxygen saturation, respiratory rate, pulse, and temperature, and also sending readings to a tablet. From a regulatory standpoint, device 1 has both FDA approval and CE marking; device 5 holds a CE mark; devices 2, 3, 4, and 6 are currently under development.

### Usability assessment

The usability assessment was completed in 3 countries with a total of 18 providers. In India, device training had to proceed prior to completion of the usability assessment due to scheduling, and in Kenya and Tanzania, one device was not available in time to assess prior to training, resulting in only 6 assessments conducted for this device in Senegal.

Device 1 was assessed by 18 users, of which 94% were “very satisfied” with the device, 83% noted it was “very easy” to use and 94% noted it would be “very useful” at their facility. Complete device use involved device setup, positioning sensor 1 on the patient, obtaining sensor 1 measurements, positioning sensor 2, and obtaining sensor 2 measurement. In round 1 of device use, 5 users experienced a critical error and 11 experienced a minor error or required a prompt to keep proceeding in the task, while in round 2, 2 users experienced critical errors, and 3 users experienced minor errors.

Device 2 was assessed by 17 users, of which 94% were “very satisfied” with the device, 94% noted it was “very easy” to use and 94% noted it would be “very useful” at their facility. Complete device use involved device setup, positioning the sensor, and obtaining measurements. In round 1, no users experienced a critical error, and 8 users experienced a minor error or required a prompt to keep proceeding in the task, while in round 2, one user experienced a critical error, and 4 users experienced a minor error.

Device 3 was assessed by 18 users, of which 89% were “very satisfied” with the device, 94% noted it was “very easy” to use, and 89% noted it would be “very useful” at their facility. Complete device use involved device setup, positioning the sensor, and obtaining measurements. In round 1, 1 user experienced a critical error, and 10 experienced a minor error or required a prompt to keep proceeding in the task, while in round 2, no users experienced critical errors, and 2 users experienced minor errors.

Device 4 was assessed by 18 users, of which 33% were “very satisfied” with the device, 28% noted it was “very easy” to use, and 67% noted it would be “very useful” at their facility. Complete device use involved device setup, positioning sensor 1 on the patient, obtaining sensor 1 measurement, positioning sensor 2, obtaining sensor 2 measurements, positioning sensor 3, and obtaining sensor 3 measurement. In round 1, 8 users experienced a critical error and 10 experienced a minor error or required a prompt to keep proceeding in the task, while in round 2, 1 user experienced critical errors, and 10 users experienced minor errors.

Device 5 was assessed by 5 users, of which 20% were “very satisfied” with the device, 0% noted it was “very easy” to use, and 60% noted it would be “very useful” at their facility. Complete device use involved device setup, positioning the sensor, and obtaining measurements. In round 1, 3 of 6 users experienced a critical error and 2 experienced a minor error or required a prompt to keep proceeding in the task, while in round 2, 1 user experienced critical errors, no users experienced minor errors, and 2 users were unable to complete the second round due to device malfunctions.

Finally, device 6 was assessed by 16 users, of which 25% were “very satisfied” with the device, 44% noted it was “very easy” to use, and 56% noted it would be “very useful” at their facility. Complete device use involved device setup, positioning sensor 1 on the patient, obtaining sensor 1 measurements, positioning sensor 2, and obtaining sensor 2 measurement. In round 1, 2 users experienced a critical error and 14 experienced a minor error or required a prompt to keep proceeding in the task, while in round 2, no users experienced critical errors, and 8 users experienced minor errors.

**Fig 4:**
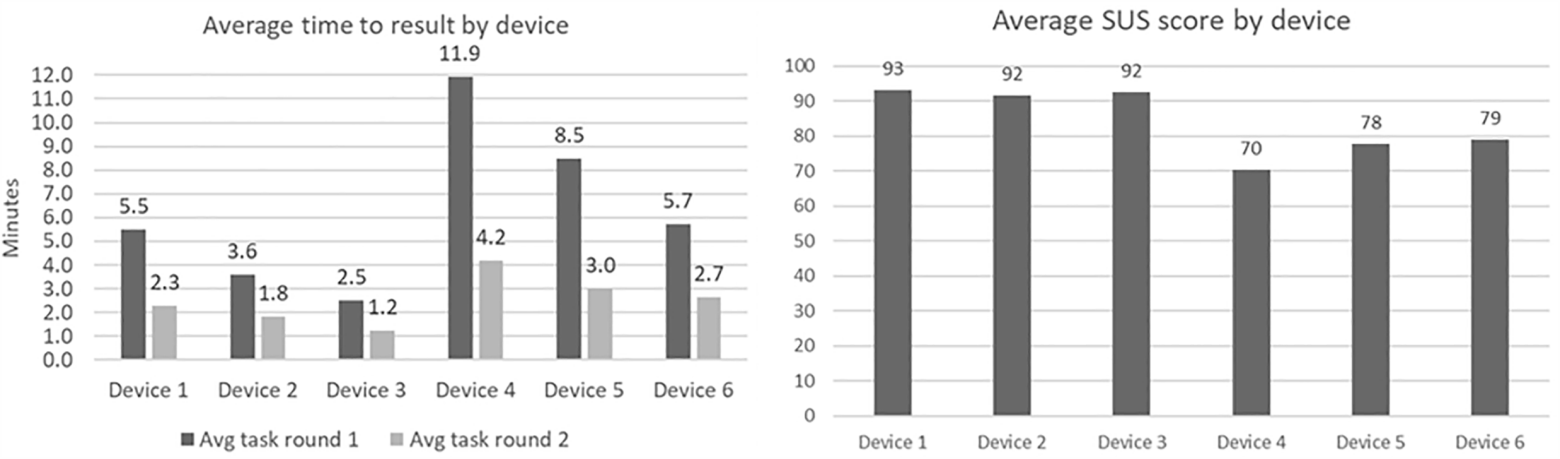
Average time to result and SUS score by device.

Fig 4 includes the average time to result and SUS score by device. Among the 6 devices, Device 3 had the shortest average time to result in round 1 at 2.5 minutes, decreasing by over 1 minute in round 2 to 1.2 minutes. Device 4 had the longest average time to result in round 1 at 11.9 minutes, decreasing by 8 minutes in round 2 to 4.2 minutes). Finally, the average SUS scores for all devices were 70 or over indicating acceptable usability. Device 1 had the highest score at 93 while Device 4 had the lowest score at 70. Full results are available in supplemental information (S1 Fig).

### Diagnostic accuracy study

Devices 1–5 were evaluated for performance. A total of 1980 participants were enrolled in the diagnostic accuracy study across the 3 countries, of which 47% were female. Most children were accompanied by their mother (79%) and common symptoms included cough (87%), fever (60%), and difficulty breathing (17%). Provider diagnoses were most often respiratory illness (83%) and fever (34%). Of the 77% (1520/1980) of participants who received treatment, 58% of the treatments were antibiotics. Rates of stunting, wasting, and anemia varied by country, as did abnormal measurements for blood oxygen, pulse, temperature, and respiratory rate. For detailed participant characteristics, see Table 2.

**Table 2:** Participant characteristics in the diagnostic accuracy study.

Demographic and physical characteristics % (n)	Overall (n = 1980)	India (n = 657)	Kenya (n = 673)	Tanzania (n = 650)
**Age Category**				
0-1 month	21.8 (432)	22.8 (150)	21.5 (145)	21.1 (137)
2-11 months	31.5 (624)	30.7 (202)	31.2 (210)	32.6 (212)
12-59 months	46.7 (924)	46.4 (305)	47.3 (318)	46.3 (301)
**Female gender**	46.6 (923)	44.1 (290)	49.6 (334)	46.0 (299)
**Relationship of caregiver to child**				
Mother only	78.8 (1560)	48.4 (318)	93.9 (632)	93.8 (610)
Mother and father	9.7 (192)	24.2 (159)	1.3 (9)	3.7 (24)
Father only	4.7 (94)	12.6 (83)	0.3 (2)	1.4 (9)
All others	6.8 (134)	14.8 (97)	4.5 (30)	1.1 (7)
**Symptoms noted at presentation**				
Cough	86.8 (1718)	69.9 (459)	91.4 (615)	99.1 (644)
Fever	59.8 (1185)	63.2 (415)	62.9 (423)	53.4 (347)
Difficulty/ Rapid breathing	17.1 (339)	3.3 (22)	39.2 (264)	8.2 (53)
Diarrhea	4.9 (98)	4.7 (31)	9.1 (61)	0.9 (6)
Vomiting	4.3 (85)	4.6 (30)	7.6 (51)	0.6 (4)
**Diagnosis received by provider**				
Respiratory	82.6 (1636)	68.8 (452)	99.6 (670)	79.1 (514)
Fever	34.4 (681)	60.1 (395)	40.1 (270)	2.5 (16)
Digestive	7.1 (140)	9.9 (65)	11.0 (74)	0.2 (1)
Dehydration	2.3 (46)	4.3 (28)	2.1 (14)	0.6 (4)
Throat infection	1.5 (29)	2.7 (18)	1.6 (11)	0.0 (0)
Malaria	0.3 (6)	0.0 (0)	0.1 (1)	0.8 (5)
Ear infection	0.3 (6)	0.3 (2)	0.6 (4)	0.0 (0)
No diagnosis received	7.0 (139)	0.6 (4)	0.0 (0)	20.8 (135)
**Treatment received**				
Antibiotic	58.1 (1151)	70.6 (465)	74.3 (500)	28.6 (186)
Bronchodilator	13.8 (274)	30.3 (199)	6.4 (43)	4.9 (32)
Dehydration	4.0 (82)	5.3 (37)	6.2 (42)	0.5 (3)
Antimalarial	0.4 (7)	0.2 (1)	0.1 (1)	0.8 (5)
No treatment received	23.2 (459)	0.6 (4)	0.6 (4)	69.4 (451)
**Stunting**				
Normal	67.4 (1335)	65.6 (431)	85.7 (577)	50.3 (327)
Moderate	13.5 (268)	14.9 (98)	10.1 (68)	15.7 (102)
Severe	15.1 (298)	19.5 (128)	3.3 (22)	22.8 (148)
No measurement	4.0 (79)	0.0 (0)	0.9 (6)	11.2 (73)
**Wasting**				
Overweight	12.4 (245)	12.5 (82)	4.6 (31)	20.3 (132)
Normal	65.9 (1305)	54.9 (361)	85.7 (577)	56.5 (367)
Moderately Wasted	8.2 (162)	13.2 (87)	6.7 (45)	4.6 (30)
Severely Wasted	6.3 (124)	13.7 (90)	2.1 (14)	3.1 (20)
No measurement	7.3 (144)	5.6 (37)	0.9 (6)	15.5 (101)
**Anemia**				
Normal	27.6 (547)	21.9 (144)	29.3 (197)	31.7 (206)
Mild	14.6 (290)	10.5 (69)	18.0 (121)	15.4 (100)
Moderate	18.6 (368)	25.9 (170)	18.6 (125)	11.2 (73)
Severe	1.5 (30)	3.3 (22)	0.9 (6)	0.3 (2)
**Not classified due to <6m	37.6 (745)	38.4 (252)	33.3 (224)	41.1 (269)
**By reference measurements**				
Abnormal blood oxygen saturation (n = 1980)	2.3 (46)	2.0 (13)	3.7 (25)	1.2 (8)
Abnormal pulse rate (n = 1980)	16.8 (332)	16.0 (105)	8.2 (55)	26.5 (172)
Abnormal temperature (n = 1062)	6.4 (127)	0.5 (3)	9.1 (61)	9.7 (63)
Abnormal respiratory rate (n = 951)	17.2 (341)	15.8 (104)	25.4 (171)	10.2 (66)

**Fig 5:**
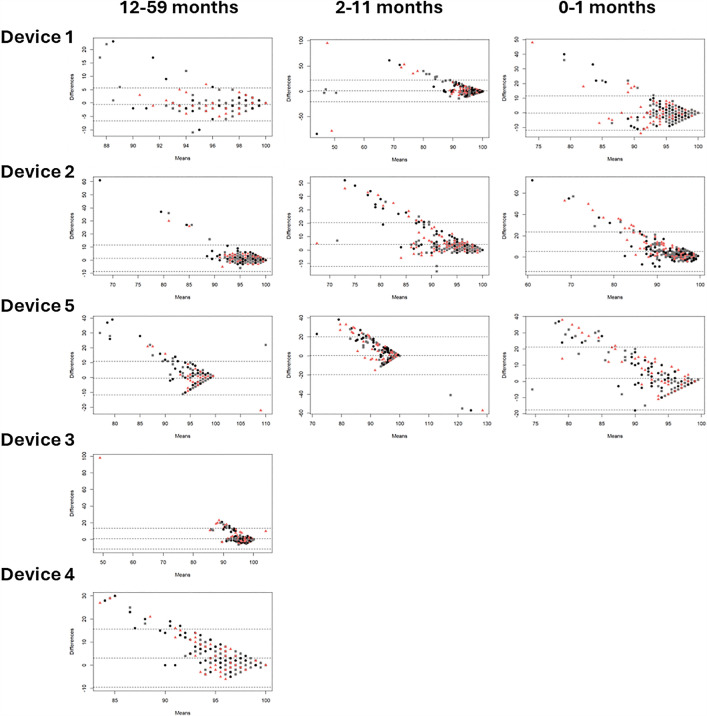
Bland Altman plots of blood oxygen saturation by device and age category.Data labels displayed represent M1 (black circle), M2 (pink triangle), and M3 (grey square).

**Fig 6:**
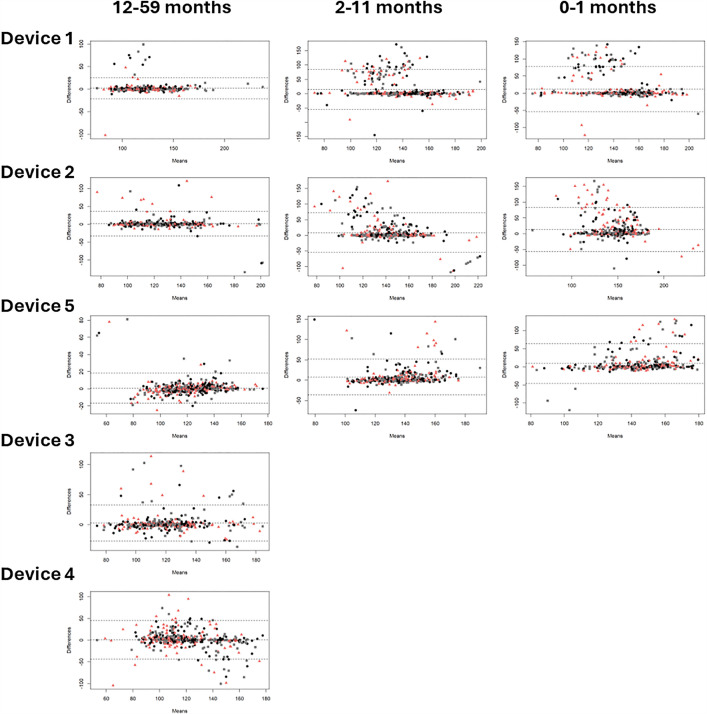
Bland Altman plots of pulse rate by device and age category.Data labels displayed represent M1 (black circle), M2 (pink triangle), and M3 (grey square).

**Fig 7:**
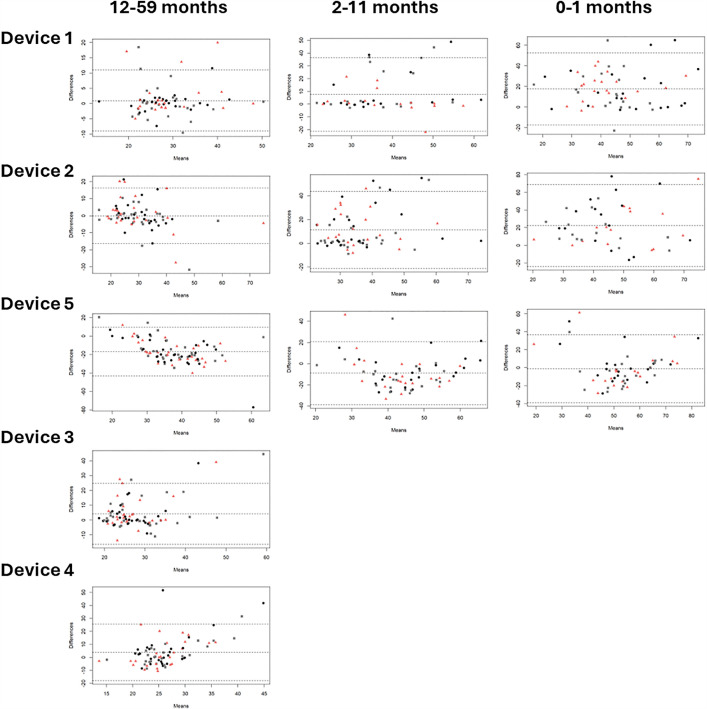
Bland Altman plots of respiratory rate by device and age category.Data labels displayed represent M1 (black circle), M2 (pink triangle), and M3 (grey square).

**Fig 8:**
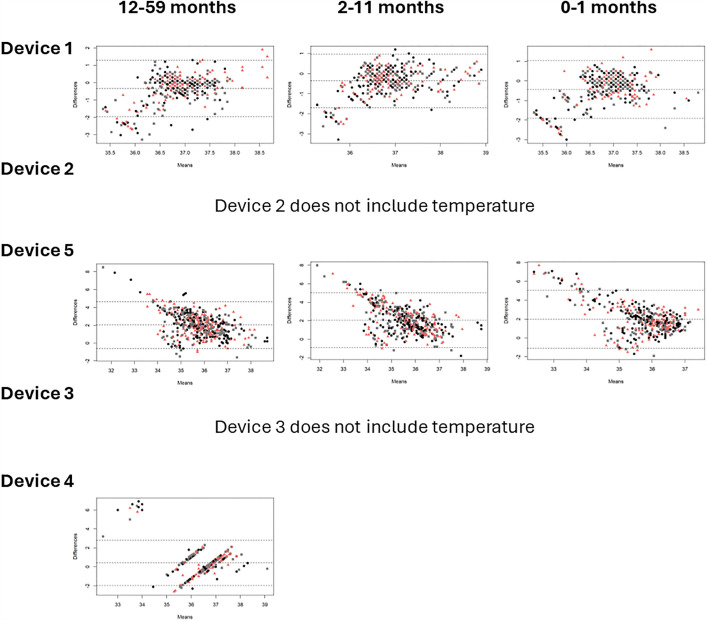
Bland Altman plots of temperature by device and age category.Data labels displayed represent M1 (black circle), M2 (pink triangle), and M3 (grey square).

**Bias:** Bland Altman plots were constructed for each clinical measurement, device, and age category showing mean differences or bias between the index and reference measurement. Fig 5 indicates that average blood oxygen saturation bias across five devices ranged from -0.49 to 3.04 (12–59 months), 0.22 to 4.12 (2–11 months), and -0.17 to 5.05 (0–1 months). Fig 6 shows pulse rate bias ranged from 0.57 to 2.23, 7.58 to 14.91, and 9.13 to 13.13 for the same age groups respectively. In Fig 7, respiratory rate bias varied from -16.68 to 4.23, -8.91 to 11.25, and -1.19 to 22.45. Finally, Fig 8 shows temperature bias from 0.34 to 2.03, -0.36 to 2.05, and -0.43 to 1.98 across these age categories.

**Error:** Mean absolute errors, which are the same as mean differences from the Bland Altman plots, are provided along with standard deviation and limits of agreement in Table 4 below. For blood oxygen saturation, pulse rate, temperature, and respiratory rate measurement errors varied by device, but varied similarly by age group across devices. Across devices, removing outliers minimally changed the magnitude of error, particularly for respiratory rate that included data cleaning for annotating videos. For detailed data, see Table 3.

**Table 3:** Mean absolute error, standard deviation, and 95% limit of agreement with and without outliers, by clinical parameter, age category, and device.

Clinical parameter	Device 1		Device 2		Device 3		Device 4		Device 5	
	MAE All (SD, 95% LOA)	MAE No outlier (SD)	MAE All (SD, 95% LOA)	MAE No outlier (SD)	MAE All (SD, 95% LOA)	MAE No outlier (SD)	MAE All (SD, 95% LOA)	MAE No outlier (SD)	MAE All (SD, 95% LOA)	MAE No outlier (SD)
**Blood oxygen saturation**										
0-1 m	0.17 (6.04, 11.84)	0.54 (3.6)	5.05 (9.48, 18.57)	2.89 (3.74)	NA	NA	NA	NA	1.73 (9.92, 19.45)	0.54 (5.34)
2-11 m	0.93 (10.91, 21.38)	0.05 (4.09)	4.12 (8.31, 16.29)	2.25 (2.89)	NA	NA	NA	NA	0.22 (10.15, 19.89)	0.63 (5.49)
12-59 m	0.49 (3.12, 6.12)	0.66 (2.46)	1.42 (5.12, 10.04)	0.83 (2.07)	1.12 (6.39, 12.52)	0.64 (3.78)	3.04 (6.41, 12.57)	2.02 (4.28)	0.27 (5.78, 11.32)	0.82 (3.52)
**Pulse rate**										
0-1 m	12.05 (33.61, 65.87)	5.57 (18.87)	13.13 (35.44, 69.45)	6.58 (18.31)	NA	NA	NA	NA	9.13 (28.19, 55.25)	5.11 (15.39)
2-11 m	14.91 (35.90, 70.36)	8.75 (23.17)	8.64 (32.34, 63.39)	6.08 (16.42)	NA	NA	NA	NA	7.58 (22.52, 44.15)	3.92 (10.52)
12-59 m	1.77 (11.87, 23.26)	1.14 (6.86)	1.45 (17.72, 34.73)	1.09 (7.55)	2.23 (15.32, 30.03)	1.21 (11.57)	1.06 (22.73, 44.56)	2.92 (16.56)	0.57 (9.01, 17.65)	0.22 (5.44)
**Temperature**										
0-1 m	0.43 (0.75, 1.47)	0.24 (0.51)	NA	NA	NA	NA	NA	NA	1.98 (1.58, 3.09)	1.74 (1.01)
2-11 m	0.36 (0.68, 1.33)	0.23 (0.5)	NA	NA	NA	NA	NA	NA	2.05 (1.52, 2.98)	1.75 (1.1)
12-59 m	0.34 (0.83, 1.63)	0.19 (0.57)	NA	NA	NA	NA	0.42 (1.22, 2.38)	0.31 (0.68)	2.03 (1.34, 2.62)	1.96 (1.07)
**Respiratory rate compared to annotation**										
0-1 m	17.70 (17.89, 35.06)	18.23 (17.97)	22.45 (23.61, 46.28)	23.77 (23.81)	NA	NA	NA	NA	1.19 (19.21, 37.66)	0.23 (21.16)
2-11 m	7.66 (14.69, 28.80)	7.66 (14.69)	11.25 (16.55, 32.44)	11.39 (16.64)	NA	NA	NA	NA	8.91 (15.16, 29.71)	8.68 (15.79)
12-59 m	0.96 (5.11, 10.01)	0.96 (5.11)	0.11 (8.33, 16.33)	0.35 (7.62)	4.23 (10.45, 20.49)	4.23 (10.45)	3.85 (11.03, 21.63)	3.24 (9.67)	16.68 (13.39, 26.24)	15.41 (11.44)

**Percent agreement:** Overall percent agreement, positive and negative percent agreement, as well as positive and negative predictive values are determined by clinical measurement, device, and age category. Percent agreement and predictive values for hypoxemia, tachycardia, fever, and fast breathing varied by device and age group, with generally high agreement for hypoxemia and tachycardia, moderate for fever, and variable for fast breathing. For full details and breakdowns, see Table 4. Because each measurement was captured 3 times in a row, except annotated respiratory rate, agreements were also evaluated by measurement attempt, which are shared in supplemental material along with fast breathing results using acoustic respiratory rate (S3 Text).

**Table 4:** Percent agreement and predictive values by clinical measurement, device, and age category (n = abnormal measurement by reference device).

	Age	Overall percent agreement (95%CI)	Positive percent agreement (95%CI)	Negative percent agreement (95%CI)	Positive predictive value (95%CI)	Negative predictive value (95%CI)
**Hypoxemia**						
**Device 1**	Overall	0.92 (0.91-0.94)	0.34 (0.20-0.51)	0.94 (0.93-0.95)	0.16 (0.09-0.26)	0.98 (0.97-0.98)
(n = 21)	0-1 m	0.91 (0.87-0.93)	0.19 (0.05-0.42)	0.94 (0.92-0.97)	0.15 (0.04-0.35)	0.96 (0.93-0.97)
(n = 17)	2-11 m	0.89 (0.85-0.92)	0.53 (0.28-0.77)	0.90 (0.87-0.93)	0.18 (0.09-0.32)	0.98 (0.96-0.99)
(n = 3)	12-59 m	0.97 (0.95-0.99)	0.33 (0.01-0.91)	0.98 (0.96-0.99)	0.09 (0.00-0.41)	1.00 (0.98-1.00)
**Device 2**	Overall	0.86 (0.84-0.88)	0.62 (0.44-0.78)	0.87 (0.85-0.89)	0.12 (0.07-0.18)	0.99 (0.98-0.99)
(n = 12)	0-1 m	0.78 (0.73-0.82)	0.50 (0.21-0.79)	0.79 (0.74-0.83)	0.07 (0.03-0.15)	0.98 (0.96-0.99)
(n = 21)	2-11 m	0.85 (0.81-0.88)	0.71 (0.48-0.89)	0.85 (0.81-0.89)	0.20 (0.12-0.31)	0.98 (0.96-0.99)
(n = 1)	12-59 m	0.96 (0.94-0.98)	0.00 (0.00-0.98)	0.96 (0.94-0.98)	0.00 (0.00-0.20)	1.00 (0.99-1.00)
**Device 3** (n = 2)	12-59 m	0.92 (0.89-0.95)	0.00 (0.00-0.84)	0.93 (0.90-0.95)	0.00 (0.00-0.12)	0.99 (0.98-1.00)
**Device 4** (n = 0)	12-59 m	0.88 (0.85-0.91)	NA	0.88 (0.85-0.91)	0.00 (0.00-0.07)	1.00 (0.99-1.00)
**Device 5**	Overall	0.86 (0.84-0.88)	0.18 (0.07-0.35)	0.88 (0.86-0.90)	0.04 (0.02-0.09)	0.97 (0.96-0.98)
(n = 15)	0-1 m	0.78 (0.73-0.82)	0.20 (0.04-0.48)	0.80 (0.76-0.85)	0.05 (0.01-0.13)	0.95 (0.92-0.98)
(n = 14)	2-11 m	0.84 (0.80-0.88)	0.21 (0.05-0.51)	0.87 (0.83-0.90)	0.06 (0.01-0.17)	0.96 (0.94-0.98)
(n = 4)	12-59 m	0.94 (0.91-0.96)	0.00 (0.00-0.60)	0.95 (0.92-0.97)	0.00 (0.00-0.15)	0.99 (0.98-1.00)
**Tachycardia**						
**Device 1**	Overall	0.93 (0.92-0.95)	0.60 (0.52-0.67)	0.98 (0.98-0.99)	0.86 (0.78-0.91)	0.94 (0.92-0.95)
**(n = 41)**	0-1 m	0.92 (0.89-0.95)	0.32 (0.18-0.48)	0.99 (0.97-1.00)	0.76 (0.50-0.93)	0.93 (0.90-0.95)
(n = 33)	2-11 m	0.92 (0.89-0.95)	0.24 (0.11-0.42)	0.98 (0.96-0.99)	0.50 (0.25-0.75)	0.94 (0.91-0.96)
(n = 82)	12-59 m	0.95 (0.93-0.97)	0.82 (0.72-0.89)	0.99 (0.97-1.00)	0.93 (0.85-0.98)	0.96 (0.93-0.98)
**Device 2**	Overall	0.93 (0.91-0.94)	0.61 (0.53-0.68)	0.98 (0.97-0.99)	0.84 (0.76-0.90)	0.94 (0.92-0.95)
(n = 42)	0-1 m	0.89 (0.86-0.92)	0.17 (0.07-0.31)	0.99 (0.97-1.00)	0.58 (0.28-0.85)	0.90 (0.87-0.93)
(n = 20)	2-11 m	0.95 (0.93-0.97)	0.40 (0.19-0.64)	0.98 (0.96-0.99)	0.50 (0.25-0.75)	0.97 (0.95-0.98)
(n = 96)	12-59 m	0.95 (0.93-0.97)	0.84 (0.76-0.91)	0.99 (0.97-1.00)	0.94 (0.87-0.98)	0.96 (0.93-0.98)
**Device 3** (n = 72)	12-59 m	0.94 (0.91-0.96)	0.63 (0.50-0.75)	0.99 (0.97-1.00)	0.88 (0.75-0.96)	0.94 (0.91-0.96)
**Device 4** (n = 86)	12-59 m	0.88 (0.84-0.91)	0.72 (0.61-0.81)	0.92 (0.88-0.94)	0.70 (0.59-0.79)	0.93 (0.89-0.95)
**Device 5**	Overall	0.93 (0.92-0.95)	0.44 (0.35-0.54)	1.00 (0.99-1.00)	0.93 (0.84-0.98)	0.93 (0.92-0.95)
(n = 26)	0-1 m	0.91 (0.88-0.94)	0.00 (0.00-0.13)	1.00 (0.98-1.00)	0.00 (0.00-0.98)	0.92 (0.88-0.95)
(n = 14)	2-11 m	0.96 (0.94-0.98)	0.00 (0.00-0.23)	1.00 (0.99-1.00)	NA	0.96 (0.94-0.98)
(n = 72)	12-59 m	0.95 (0.93-0.97)	0.75 (0.63-0.84)	0.99 (0.98-1.00)	0.95 (0.85-0.99)	0.95 (0.93-0.97)
**Fever**						
**Device 1**	Overall	0.87 (0.85-0.89)	0.70 (0.62-0.77)	0.90 (0.88-0.91)	0.48 (0.41-0.54)	0.96 (0.94-0.97)
(n = 33)	0-1 m	0.87 (0.83-0.90)	0.67 (0.48-0.82)	0.88 (0.85-0.91)	0.33 (0.22-0.45)	0.97 (0.94-0.98)
(n = 61)	2-11 m	0.90 (0.87-0.93)	0.77 (0.65-0.87)	0.92 (0.89-0.95)	0.61 (0.49-0.72)	0.96 (0.94-0.98)
(n = 57)	12-59 m	0.85 (0.82-0.89)	0.63 (0.49-0.76)	0.89 (0.85-0.92)	0.47 (0.35-0.58)	0.94 (0.91-0.96)
**Device 4** (n = 66)	12-59 m	0.85 (0.82-0.89)	0.08 (0.03-0.17)	1.00 (0.98-1.00)	0.83 (0.36-1.00)	0.85 (0.82-0.89)
**Device 5**	Overall	0.86 (0.84-0.88)	0.07 (0.04-0.12)	1.00 (0.99-1.00)	0.82 (0.57-0.96)	0.86 (0.84-0.88)
(n = 49)	0-1 m	0.88 (0.84-0.91)	0.00 (0.00-0.07)	1.00 (0.99-1.00)	NA	0.88 (0.84-0.91)
(n = 52)	2-11 m	0.88 (0.85-0.91)	0.06 (0.01-0.16)	0.99 (0.98-1.00)	0.60 (0.15-0.95)	0.89 (0.85-0.92)
(n = 96)	12-59 m	0.82 (0.78-0.85)	0.11 (0.06-0.20)	1.00 (0.99-1.00)	0.92 (0.62-1.00)	0.82 (0.78-0.85)
**Fast breathing (by annotation)**						
**Device 1**	overall	0.75 (0.68-0.81)	0.42 (0.30-0.54)	0.96 (0.90-0.99)	0.86 (0.70-0.95)	0.72 (0.65-0.79)
(n = 37)	0-1 m	0.54 (0.41-0.67)	0.30 (0.16-0.47)	0.95 (0.77-1.00)	0.92 (0.62-1.00)	0.45 (0.30-0.60)
(n = 16)	2-11 m	0.78 (0.63-0.88)	0.38 (0.15-0.65)	0.97 (0.84-1.00)	0.86 (0.42-1.00)	0.76 (0.61-0.88)
(n = 4)	12-35 m	0.88 (0.70-0.98)	0.50 (0.07-0.93)	0.95 (0.77-1.00)	0.67 (0.09-0.99)	0.91 (0.72-0.99)
(n = 14)	36-59 m	0.91 (0.79-0.97)	0.79 (0.49-0.95)	0.95 (0.83-0.99)	0.85 (0.55-0.98)	0.92 (0.80-0.98)
**Device 2**	overall	0.70 (0.63-0.76)	0.40 (0.28-0.52)	0.88 (0.81-0.94)	0.67 (0.51-0.81)	0.71 (0.63-0.78)
(n = 28)	0-1 m	0.44 (0.30-0.60)	0.21 (0.08-0.41)	0.82 (0.57-0.96)	0.67 (0.30-0.93)	0.39 (0.23-0.57)
(n = 16)	2-11 m	0.81 (0.70-0.89)	0.25 (0.07-0.52)	0.98 (0.90-1.00)	0.80 (0.28-0.99)	0.81 (0.69-0.90)
(n = 3)	12-35 m	0.89 (0.72-0.98)	0.67 (0.09-0.99)	0.92 (0.74-0.99)	0.50 (0.07-0.93)	0.96 (0.79-1.00)
(n = 24)	36-59 m	0.72 (0.58-0.83)	0.71 (0.49-0.87)	0.72 (0.53-0.87)	0.68 (0.46-0.85)	0.75 (0.55-0.89)
**Device 3**	overall	0.83 (0.74-0.90)	0.54 (0.33-0.73)	0.95 (0.87-0.99)	0.82 (0.57-0.96)	0.83 (0.73-0.91)
(n = 4)	12-35 m	0.88 (0.69-0.97)	0.25 (0.01-0.81)	1.00 (0.84-1.00)	1.00 (0.03-1.00)	0.88 (0.68-0.97)
(n = 0)	36-59 m	0.81 (0.69-0.90)	0.59 (0.36-0.79)	0.92 (0.80-0.98)	0.81 (0.54-0.96)	0.80 (0.66-0.91)
**Device 4**	overall	0.74 (0.64-0.83)	0.18 (0.05-0.40)	0.95 (0.86-0.99)	0.57 (0.18-0.90)	0.76 (0.65-0.85)
(n = 2)	12-35 m	0.89 (0.67-0.99)	0.00 (0.00-0.84)	1.00 (0.80-1.00)	NA	0.89 (0.67-0.99)
(n = 20)	36-59 m	0.70 (0.57-0.81)	0.20 (0.06-0.44)	0.93 (0.81-0.99)	0.57 (0.18-0.90)	0.71 (0.58-0.83)
**Device 5**	overall	0.47 (0.40-0.54)	0.86 (0.75-0.93)	0.28 (0.21-0.36)	0.36 (0.29-0.44)	0.80 (0.67-0.90)
(n = 33)	0-1 m	0.54 (0.41-0.67)	0.79 (0.61-0.91)	0.23 (0.09-0.44)	0.57 (0.41-0.71)	0.46 (0.19-0.75)
(n = 13)	2-11 m	0.50 (0.37-0.63)	0.77 (0.46-0.95)	0.43 (0.30-0.58)	0.25 (0.13-0.41)	0.88 (0.70-0.98)
(n = 6)	12-35 m	0.40 (0.24-0.58)	1.00 (0.54-1.00)	0.28 (0.13-0.47)	0.22 (0.09-0.42)	1.00 (0.63-1.00)
(n = 15)	36-59 m	0.39 (0.26-0.53)	1.00 (0.78-1.00)	0.15 (0.06-0.31)	0.31 (0.19-0.46)	1.00 (0.54-1.00)

## Discussion

This multi-dimensional research generated findings on product validation, diagnostic accuracy and insights on market entry. Through the usability assessment, all study devices showed good usability with few observed user errors, high perceived satisfaction and usefulness, and high system usability scores though devices with more procedures had more user challenges, particularly involving connected capabilities. In the diagnostic accuracy study involving 1980 participants, percent agreements for hypoxemia, tachycardia, and fever among all devices demonstrated greater than 80% agreement for all age categories; respiratory rate measurement demonstrated more variability in overall percent agreement across devices (39–91%). In general, mean absolute error across the 5 devices was less among the older age categories compared to younger age category for all clinical measurements. Diverse inputs from stakeholders and manufacturers defined key attributes of next generation multimodal devices including high interest in respiratory rate, temperature and hemoglobin, and features like providing a quick result, long battery life, and applicability for all ages.

Multiple indicators of accuracy were included in this analysis, offering a more nuanced assessment of device performance and addressing the varying priorities of stakeholder groups. Starting with the more straightforward, overall agreement for all devices was over 85%, except in one subcategory, when measuring hypoxemia, tachycardia, and fever. And while positive percent agreement and positive predictive values were low, largely due to the fewer number of cases (n = 46 for hypoxemia, n = 332 for tachycardia, n = 127 for fever, and n = 341 for fast breathing), negative percent agreement and negative predictive value were higher and may be useful for all clinical measurements including detection of fast breathing. Additionally, overall agreement appeared to improve with repeated measures (table in supplemental information). Given that fast breathing is measured only 10–20% of the time that it should be measured, the negative predictive value of a multimodal device may assist providers in determining that a patient does not have fast breathing, even if a positive detection requires further screening. Additionally, these noninvasive devices provide results in 1–4 minutes, according to the usability results, and may allow repeated measurements of positive results to be a practical approach for improving accuracy in operational workflows. More research is needed to assess the performance of these devices if used in a two or three test strategy, or with consideration for pre-test probability, as it also adds complexity [51].

Giving a more nuanced interpretation, the Bland Altman plots show varying patterns of data spread across clinical measurements. For blood oxygen saturation, the funnel shape suggests measurement error is greater at lower values, and the bias is overestimating the true value in lower values. For pulse rate, the bias is more consistent across the range of values, but where error occurs it is overestimating the true value. For respiratory rate, the pattern for device 1 suggests consistent bias across the range of values but more overestimation in the younger age categories, while device 5 has a pattern suggesting consistent bias with more underestimation in the older age categories, and devices 3 and 4 show more overestimation at higher values. Finally, for temperature, the device 1 pattern suggests consistent underestimation, device 5 suggests overestimation at lower values and underestimation and higher values, and device 4 has a distinct pattern likely due to the contact thermometer taking more time to develop a stable reading so the 3 measurement attempts varied in a consistent manner. From a clinical care perspective, overestimation of blood oxygen saturation, and underestimation of pulse rate, respiratory rate, and temperature are more dangerous biases, particularly around the thresholds for abnormal, as false negatives would result in children being missed who may be more sick and could benefit from further care sooner. This level of detail provides information useful for device development and ongoing improvement, especially as these devices are at different stages of product development, including those already on the market and approved by regulatory authorities, those design-locked but not yet available commercially, and those seeking to expand product claims to younger age categories. The form factors were also highly diverse with traditional handheld designs, finger clip designs, and connected designs that required a tablet to operate. Overall, across devices and clinical measurements, performance was often best in the oldest age category, similar to other recent studies [38,52,53]. Larger digits and minimal or more controlled movements make it easier for medical devices to capture more accurate data.

From a user and market perspective, the usabilty assessment highlighted that fewer device procedures improved usability while connected capabilities increased user challenge. Despite these challenges, repeated use led to fewer errors, demonstrating that even complex devices could be learned over time.. Throughout this research, stakeholders and providers expressed high interest in devices with expanded feature sets, particularly the integration of temperature measurement, which sometimes was valued over anemia assessment despite thermometers being affordable and accessible, reflecting evolving needs since the Covid-19 pandemic increased attention toward non-contact thermometers [54]. Incorporating such features into multimodal diagnostic tools may enhance the user experience and clinical utility, especially if development involves diverse stakeholder input to ensure relevance and adoption [55]. However, as devices become more advanced and capable of capturing additional clinical parameters such as hemoglobin and blood pressure through PPG-derived measurements [24,56–60], the mental workload on providers also increases. This underscores the importance of supportive innovations, such as AI-driven decision support tools and automated documentation systems, to help providers efficiently interpret and act on the growing volume of health data. Balancing feature expansion with usability and workflow integration is therefore essential to ensure these technologies meet both user needs and market demand, ultimately optimizing the provider experience [61].

Study limitations included complicated device setup, possibly excessive performance measures, and an assessment of perceived demand for next-generation multimodal devices that may differ from actual demand. In the diagnostic accuracy study, managing the complexity of device setup and conducting simultaneous measurements across multiple instruments posed significant challenges, particularly in maintaining a calm and cooperative environment for the children involved. Though this could lead to spurious readings, extensive practice with the technology and daily data review ensured high quality data collection. Additionally, a sensitivity analysis was performed to understand if outlier measurements had impacted the results, which showed little change. While we included a broad range of diagnostic performance measures to provide a comprehensive assessment, this may have introduced complexity that could obscure findings or reduce interpretability for some audiences. However, the different metrics are of varying utility for different audiences, such as manufacturers, health care providers, or policymakers, and therefore sharing the nuance was prioritized over potential information overload. Finally, TPPs play a vital role in articulating requirements and aligning the global health community and industry stakeholders with product class needs. Nevertheless, our study reflects only perceived demand; generating evidence of demonstrated demand is essential to triangulate and ensure new products are appropriately positioned for market entry.

In conclusion, this study generated key evidence around the performance of current multimodal pulse oximeter devices across a range of form factors and with varying product attributes. The results are presented in a manufacturer-neutral manner to account for continuing product improvements throughout different stages of product development. While all devices were usable with training and consistent practice, less frequent use may challenge provider recall and ease of use, resulting in diminishing adherence over time in their already brief consultation visits. Moreover, rising stakeholder interest in feature sets should be considered alongside increasing requirements for providers to implement further measurements or assessments, since this could overburden health professionals if essential features such as integration and connectivity are not prioritized as well. The manufacturer and research community have a critical opportunity to leverage emerging technologies to improve the experience of providers, and as a result, the experience of patients.

## Supporting information

S1 Checklist Completed STARD checklist. (DOCX)

S2 Checklist Inclusivity in global research questionnaire. (DOCX)

S1 Text Development of a target product profile for multimodal PO devices. (DOCX)

S2 Text Specifications of multimodal PO devices by product attribute, highlighting good (green) to poor (red) alignment to minimum requirements of the target product profile. (DOCX)

S3 Text Overall percent agreement by measurement attempt (M1-3) and age category. (DOCX)

S1 Fig Detailed usability results by device. (DOCX)
