# Supplementary material for: Multimodal pulse oximeters to support the integrated management of childhood illnesses: A usability and diagnostic accuracy assessment from a multi-country hybrid type 2 study
Source: PLOS Glob Public Health. 2026 Mar 26;6(3):e0004655. doi: 10.1371/journal.pgph.0004655 (PMC13020799; doi:10.1371/journal.pgph.0004655)
Supplement: S1 Text — (DOCX) [file pgph.0004655.s003.docx]

**S1 Text. Development of a target product profile for multimodal PO devices.**

**Methods:** To define requirements of next generation multimodal PO devices, a target product profile (TPP) was developed through a multi-phase process. The TPP communicates requirements for optimal and minimum product attributes to align the global health community and manufacturers with the needs for the product class. To start, PATH reviewed the literature and conducted a series of expert interviews to develop a draft TPP for multimodal devices in primary care settings. The literature review focused on relevant publications addressing pulse oximetry use in primary care settings, as well as measurement of respiratory rate, temperature, and hemoglobin in similar contexts. Policy documents and guidelines were also assessed, along with previous partner TPPs on automated respiratory rate devices, dual PO and respiratory rate devices, as well as hemoglobinometers. Experts were identified for stakeholder interviews through current partnerships, authorship on key peer-reviewed publications, and snowball sampling. At least two researchers conducted or reviewed each interview using a semi-structured interview guide. Interview questions focused on the main challenges with managing and diagnosing ill children at the primary care level, availability of existing tools to measure vital signs and other clinical parameters including advantages and disadvantages, context of use considerations related to the facility, the environment, and the health professional, and other potential use cases for multimodal devices. A draft TPP was generated summarizing the findings.

Workshops were conducted in Kenya, Tanzania, and Senegal to obtain feedback on the draft TPP from a national stakeholder perspective. The workshops involved a series of interactive, human-centered design activities, including small group based and individual data collection. They were conducted in person (Senegal, Tanzania) or virtually (Kenya), and participants were invited based on their experience in child health research, clinical practice at the primary care level, and national guideline development and implementation involving the Integrated Management of Childhood Illnesses (IMCI). Activities were conducted to generate insights on the patient journey of a sick child at the PHC level, to prioritize clinical measurements and features, and to explore opportunities for future multimodal devices using journey mapping, priority ranking, and I like/I wish/I wonder methodologies. The draft TPP was shared prior to the workshop and participants were asked to review in advance.

An online survey was conducted to assess agreement on the minimum and optimal requirements for 23 product attributes. The survey was shared through partner networks by the TIMCI project, CHAI, UNICEF, Every Breath Counts, and the International Pediatric Association. For each requirement, respondents were asked to indicate whether they “agree”, “mostly agree”, “neither agree or disagree”, “mostly disagree”, “fully disagree”, or “other (do not have expertise to comment)”. A predefined agreement threshold of at least 60% selecting “agree”, “mostly agree”, or “neither agree or disagree” was set. Other areas of feedback requested were ranking clinical measurements, gauging willingness to pay, prioritizing device features, and identifying reference standards for the primary clinical parameters. Finally, select manufacturers (n=7) were engaged in a discussion where the draft TPP was shared, and challenges and opportunities with key product attributes were explored. Notes were recorded by notetakers during the workshop and survey results were analyzed in Microsoft Excel.

**Results:** In total 39 stakeholders participated in the TPP workshops in Kenya, Tanzania, and Senegal from October to December 2020. Workshop participants ranked clinical measurements from most to least important, and following oxygen saturation, the priority order based on weighted average was respiratory rate (4.18), pulse rate (3.95), temperature (3.54), hemoglobin (2.23), then blood pressure (2.03). The 3 most important product attributes identified were “provides a result in less than 1 minute”, “has a battery life of at least 12 hours, and is rechargeable”, and “can be used with all ages, from birth onward”. The three least important product attributes were “capable of being a connected device”, “requires less than 1 hour to train users”, and “the shelf life of the device is at least 7 years”.

Similar questions were included in the global consensus survey, where respondents (n=43) were mostly clinicians and researchers (65%), representing 15 countries of which 56% were high-income countries. Survey respondents ranked clinical measurements from most to least important, and following oxygen saturation, the priority order based on weighted average was respiratory rate (3.69), hemoglobin (2.97), pulse rate (2.94), blood pressure (2.81), then temperature (2.58). The 3 most important product attributes identified were “requires no calibration and minimal maintenance over time”, “has a battery life of at least 12 hours, and is rechargeable”, and “can be used with all ages, from birth onward”. The three least important product attributes were “capable of being a connected device”, “requires less than 1 hour to train users”, and “includes a user interface that provides decision support”. Compared to a stand-alone pulse oximeter, survey respondents indicated they were most willing to pay more for hemoglobin, followed by respiratory rate, blood pressure, then temperature. Additional desired clinical measurements included ECG, capnography, and a point of care sepsis biomarker. For the 23 product attributes assessed, respondent agreement to the minimum and optimal product attributes ranged from 69-100% achieving the pre-defined threshold of at least 60% agreement across all attributes. At a threshold of 80%, only 2 attributes were below the level of agreement, indicating differing perspectives among respondents: minimum time to result, and minimum instrumentation. Respondents also shared comments for continual improvement.

Manufacturer feedback on the drafted TPP noted the resources as a “helpful guiding document for innovators” and “roadmap for future products”. While some manufacturers wanted the TPP to translate into purchase agreements, it was noted as a tool for global procurement platforms to guide product selection for catalogues, especially as newer products are released. Manufacturers provided input on newer clinical measurements including temperature and noninvasive hemoglobin. For temperature, there was recent interest from users due to Covid19, however integrating temperature into a handheld device may be challenging and expensive for companies that do not currently have the technology. The suggestion was made to decrease temperature accuracy range to 0.3 C to align with ISO standards and specify where and how measurement is taken (infrared, contact, skin, core, etc.). For noninvasive hemoglobin, manufacturers noted mixed interest from stakeholders, related to intended use and patient population.

The final TPP is accessible at <https://www.path.org/our-impact/resources/>multimodal-pulse-oximeter-tpp.

*Document. Multimodal Device: Target Product Profile; Diagnostic Instrument: Noninvasive multimodal device to measure oxygen saturation, pulse rate, respiratory rate, and additional clinical parameters as available such as, temperature, hemoglobin, and blood pressure.*
