# Supplementary material for: Multimodal pulse oximeters to support the integrated management of childhood illnesses: A usability and diagnostic accuracy assessment from a multi-country hybrid type 2 study
Source: PLOS Glob Public Health. 2026 Mar 26;6(3):e0004655. doi: 10.1371/journal.pgph.0004655 (PMC13020799; doi:10.1371/journal.pgph.0004655)
Supplement: S2 Text — (DOCX) [file pgph.0004655.s004.docx]

**S2 Text. Specifications of multimodal PO devices by product attribute, highlighting good (green) to poor (red) alignment to minimum requirements of the target product profile.**

| **Product Attributes** | **Device 1** | **Device 2** | **Device 3** | **Device 4** | **Device 5** | **Device 6** |
| --- | --- | --- | --- | --- | --- | --- |
| Measurement mode | Spot check and continuous | Spot check and continuous | Spot check only | Spot check only | Spot check and continuous | Spot check only |
| Clinical measurements | PO, PR, RR (pleth), temp | PO, PR, RR (pleth) | PO, PR, RR (pleth) | PO, PR, RR (ECG), temp, BP, ECG, HRV, blood glucose | PO, PR, RR (pleth), temp | PO, PR, RR (pleth), Hb |
| Age range | Adults and children | Adults and children | Adults only, children pending | Adults only | Adults and children | Adults and children |
| Ages assessed in DA study | 0-1, 2-11, and 12-59 months (n=150 per country total) | 0-1, 2-11, and 12-59 months (n=150 per country total) | 12-59 months (n=50 per country total) | 12-59 months (n=50 per country total) | 0-1, 2-11, and 12-59 months (n=150 per country total) | 2-11 and 12-59 months (n=100 per country total) |
| Accuracy (ARMS): Pulse oximetry, heart rate, and respiratory rate, as measured through PATH benchtop testing | 0.37%, 0.52 bpm, 0.89 breaths/min | 1.00%, 0.00 bpm, 0.77 breaths/min | 0.68%, 0.00 bpm, 0.77 breaths/min | 1.21%, 2.76 bpm, 2.65 breaths/min | 0.45%, 0.63 bpm, 1.9 breaths/min | NA |
| Time to result, as measured through PATH benchtop testing | <1 minute | <1 minute | <1 minute | <1 minute | PO, PR, RR <1 minute  Temp ~10-20 minutes | NA |
| Operating environment | 10C-40C, 10%-95% humidity | 0-40C, 15-85% humidity | -5-40C, 10-90% humidity | -20C-50C, humidity not available | 15C-35C, 30%-75% humidity | 32° - 95°F (0° - 35°C) |
| Instrumentation (fingertip, handheld, other? ) | Handheld with finger clip attached by cord, device displays measurements | Handheld with finger clip attached by cord, device displays measurements | Fingertip clip, device displays measurements | Other- patient holds device in palm of hand, device connects to tablet by Bluetooth to display measurements | Other- forehead band, device connects to tablet by Bluetooth to display measurements | Other- patient places finger or toe over camera, phone displays measurements |
| Consumables | Reusable for children/adults; neonatal probe requires disposable wrap | Reusable | Reusable | Reusable except for blood test strips | Reusable | Reusable |
| Power requirements | Rechargeable battery with adapter | Rechargeable battery with adapter | Can use rechargeable batteries (not provided) | Rechargeable battery with adapter | Rechargeable battery with adapter | Rechargeable battery with adapter |
| Cost (**TPP price has not yet been determined.) | TBD | Indicative $170-200 | Indicative $150-200 | <$0.50 per patient; subscription based | Indicative $250 | TBD  (phone; downloadable application) |
| Regulatory status | FDA approved, CE marked | In development | In development | In development | CE marked | In development |
