# Supplementary material for: Multimodal pulse oximeters to support the integrated management of childhood illnesses: A usability and diagnostic accuracy assessment from a multi-country hybrid type 2 study
Source: PLOS Glob Public Health. 2026 Mar 26;6(3):e0004655. doi: 10.1371/journal.pgph.0004655 (PMC13020799; doi:10.1371/journal.pgph.0004655)
Supplement: S3 Text — (DOCX) [file pgph.0004655.s005.docx]

**S3 Text.**

**Acoustic respiratory rate:** The percent agreement between the two reference standards for respiratory rate varies by age category: 51% (0-1 month), 72% (2-11 months), 83% (12-35 months), and 77% (36-59 months). As shared in the results section, overall percent agreements for fast breathing by annotation ranged from 47% to 83%, and for the 36-59 month group, percent agreements were 91%, 72%, 81%, 70%, and 39%, respectively for Devices 1 to 5. For all age categories, Device 1 percent agreement for respiratory rate with annotated reference was 54%, 78%, 88%, and 91% for ages 0-1 month, 2-11 months, 12-35, and 36-59 months respectively. Device 2 percent agreement for respiratory rate with annotated reference was 44%, 81%, 89%, and 72% for ages 0-1 month, 2-11 months, 12-35, and 36-59 months respectively. Device 5 percent agreement for respiratory rate with annotated reference was 54%, 50%, 40%, and 39% for ages 0-1 month, 2-11 months, 12-35, and 36-59 months respectively. In comparison, assessment of fast breathing by acoustic method demonstrated similar overall percent agreements from 39% to 83% across devices, and for the 36-59 month group, percent agreements were 80%, 69%, 82%, 77%, and 30%, respectively for Devices 1 to 5.

**Percent agreement and predictive values for fast breathing using acoustic and video annotated respiratory rate by device and age category.**

|  | Age | Overall percent agreement (95%CI) | Positive percent agreement (95%CI) | Negative percent agreement (95%CI) | Positive predictive value (95%CI) | Negative predictive value (95%CI) |  |
| --- | --- | --- | --- | --- | --- | --- | --- |
| Fast breathing (by acoustic) | | | | | | | |
| Device 1 | overall | 0.82 (0.79-0.85) | 0.48 (0.41-0.56) | 0.90 (0.88-0.92) | 0.55 (0.47-0.63) | 0.88 (0.85-0.90) |  |
|  | 0-1 m | 0.77 (0.72-0.82) | 0.29 (0.18-0.42) | 0.90 (0.85-0.93) | 0.42 (0.27-0.59) | 0.83 (0.77-0.87) |  |
|  | 2-11 m | 0.89 (0.84-0.92) | 0.43 (0.29-0.59) | 0.97 (0.94-0.99) | 0.71 (0.51-0.87) | 0.90 (0.86-0.94) |  |
|  | 12-35 m | 0.88 (0.80-0.94) | 0.56 (0.21-0.86) | 0.92 (0.84-0.97) | 0.42 (0.15-0.72) | 0.95 (0.88-0.99) |  |
|  | 36-59 m | 0.80 (0.74-0.85) | 0.72 (0.59-0.83) | 0.83 (0.76-0.88) | 0.61 (0.48-0.72) | 0.89 (0.83-0.94) |  |
| Device 2 | overall | 0.81 (0.79-0.84) | 0.40 (0.32-0.49) | 0.88 (0.86-0.90) | 0.36 (0.28-0.44) | 0.90 (0.88-0.92) |  |
|  | 0-1 m | 0.81 (0.76-0.85) | 0.28 (0.15-0.44) | 0.89 (0.84-0.92) | 0.26 (0.14-0.42) | 0.89 (0.85-0.93) |  |
|  | 2-11 m | 0.89 (0.85-0.92) | 0.24 (0.11-0.42) | 0.96 (0.93-0.97) | 0.35 (0.16-0.57) | 0.93 (0.89-0.95) |  |
|  | 12-35 m | 0.85 (0.78-0.90) | 0.13 (0.02-0.40) | 0.93 (0.87-0.96) | 0.17 (0.02-0.48) | 0.91 (0.84-0.95) |  |
|  | 36-59 m | 0.69 (0.62-0.75) | 0.67 (0.53-0.80) | 0.69 (0.62-0.77) | 0.43 (0.32-0.54) | 0.86 (0.79-0.92) |  |
| Device 3 | overall | 0.83 (0.79-0.86) | 0.57 (0.45-0.68) | 0.89 (0.85-0.92) | 0.56 (0.44-0.67) | 0.90 (0.86-0.93) |  |
|  | 12-35 m | 0.85 (0.78-0.90) | 0.28 (0.10-0.53) | 0.93 (0.87-0.97) | 0.36 (0.13-0.65) | 0.90 (0.84-0.95) |  |
|  | 36-59 m | 0.82 (0.77-0.87) | 0.71 (0.58-0.83) | 0.86 (0.80-0.90) | 0.61 (0.48-0.72) | 0.91 (0.85-0.95) |  |
| Device 4 | overall | 0.81 (0.76-0.85) | 0.17 (0.07-0.30) | 0.93 (0.89-0.95) | 0.29 (0.13-0.49) | 0.86 (0.82-0.90) |  |
|  | 12-35 m | 0.92 (0.84-0.97) | 0.00 (0.00-0.41) | 1.00 (0.95-1.00) | NA | 0.92 (0.84-0.97) |  |
|  | 36-59 m | 0.77 (0.71-0.82) | 0.20 (0.09-0.35) | 0.90 (0.84-0.93) | 0.29 (0.13-0.49) | 0.84 (0.78-0.89) |  |
| Device 5 | overall | 0.39 (0.36-0.42) | 0.91 (0.86-0.95) | 0.30 (0.26-0.33) | 0.19 (0.16-0.22) | 0.95 (0.92-0.97) |  |
|  | 0-1 m | 0.42 (0.36-0.48) | 0.80 (0.66-0.91) | 0.34 (0.28-0.41) | 0.20 (0.15-0.27) | 0.89 (0.81-0.95) |  |
|  | 2-11 m | 0.47 (0.41-0.53) | 0.95 (0.74-1.00) | 0.44 (0.38-0.50) | 0.11 (0.06-0.16) | 0.99 (0.95-1.00) |  |
|  | 12-35 m | 0.37 (0.29-0.46) | 0.95 (0.74-1.00) | 0.28 (0.20-0.37) | 0.18 (0.11-0.26) | 0.97 (0.84-1.00) |  |
|  | 36-59 m | 0.30 (0.24-0.36) | 0.98 (0.90-1.00) | 0.09 (0.06-0.15) | 0.25 (0.19-0.31) | 0.94 (0.73-1.00) |  |
| Fast breathing (by annotation) | | | | | | | |
| Device 1 | overall | 0.75 (0.68-0.81) | 0.42 (0.30-0.54) | 0.96 (0.90-0.99) | 0.86 (0.70-0.95) | 0.72 (0.65-0.79) |  |
|  | 0-1 m | 0.54 (0.41-0.67) | 0.30 (0.16-0.47) | 0.95 (0.77-1.00) | 0.92 (0.62-1.00) | 0.45 (0.30-0.60) |  |
|  | 2-11 m | 0.78 (0.63-0.88) | 0.38 (0.15-0.65) | 0.97 (0.84-1.00) | 0.86 (0.42-1.00) | 0.76 (0.61-0.88) |  |
|  | 12-35 m | 0.88 (0.70-0.98) | 0.50 (0.07-0.93) | 0.95 (0.77-1.00) | 0.67 (0.09-0.99) | 0.91 (0.72-0.99) |  |
|  | 36-59 m | 0.91 (0.79-0.97) | 0.79 (0.49-0.95) | 0.95 (0.83-0.99) | 0.85 (0.55-0.98) | 0.92 (0.80-0.98) |  |
| Device 2 | overall | 0.70 (0.63-0.76) | 0.40 (0.28-0.52) | 0.88 (0.81-0.94) | 0.67 (0.51-0.81) | 0.71 (0.63-0.78) |  |
|  | 0-1 m | 0.44 (0.30-0.60) | 0.21 (0.08-0.41) | 0.82 (0.57-0.96) | 0.67 (0.30-0.93) | 0.39 (0.23-0.57) |  |
|  | 2-11 m | 0.81 (0.70-0.89) | 0.25 (0.07-0.52) | 0.98 (0.90-1.00) | 0.80 (0.28-0.99) | 0.81 (0.69-0.90) |  |
|  | 12-35 m | 0.89 (0.72-0.98) | 0.67 (0.09-0.99) | 0.92 (0.74-0.99) | 0.50 (0.07-0.93) | 0.96 (0.79-1.00) |  |
|  | 36-59 m | 0.72 (0.58-0.83) | 0.71 (0.49-0.87) | 0.72 (0.53-0.87) | 0.68 (0.46-0.85) | 0.75 (0.55-0.89) |  |
| Device 3 | overall | 0.83 (0.74-0.90) | 0.54 (0.33-0.73) | 0.95 (0.87-0.99) | 0.82 (0.57-0.96) | 0.83 (0.73-0.91) |  |
|  | 12-35 m | 0.88 (0.69-0.97) | 0.25 (0.01-0.81) | 1.00 (0.84-1.00) | 1.00 (0.03-1.00) | 0.88 (0.68-0.97) |  |
|  | 36-59 m | 0.81 (0.69-0.90) | 0.59 (0.36-0.79) | 0.92 (0.80-0.98) | 0.81 (0.54-0.96) | 0.80 (0.66-0.91) |  |
| Device 4 | overall | 0.74 (0.64-0.83) | 0.18 (0.05-0.40) | 0.95 (0.86-0.99) | 0.57 (0.18-0.90) | 0.76 (0.65-0.85) |  |
|  | 12-35 m | 0.89 (0.67-0.99) | 0.00 (0.00-0.84) | 1.00 (0.80-1.00) | NA | 0.89 (0.67-0.99) |  |
|  | 36-59 m | 0.70 (0.57-0.81) | 0.20 (0.06-0.44) | 0.93 (0.81-0.99) | 0.57 (0.18-0.90) | 0.71 (0.58-0.83) |  |
| Device 5 | overall | 0.47 (0.40-0.54) | 0.86 (0.75-0.93) | 0.28 (0.21-0.36) | 0.36 (0.29-0.44) | 0.80 (0.67-0.90) |  |
|  | 0-1 m | 0.54 (0.41-0.67) | 0.79 (0.61-0.91) | 0.23 (0.09-0.44) | 0.57 (0.41-0.71) | 0.46 (0.19-0.75) |  |
|  | 2-11 m | 0.50 (0.37-0.63) | 0.77 (0.46-0.95) | 0.43 (0.30-0.58) | 0.25 (0.13-0.41) | 0.88 (0.70-0.98) |  |
|  | 12-35 m | 0.40 (0.24-0.58) | 1.00 (0.54-1.00) | 0.28 (0.13-0.47) | 0.22 (0.09-0.42) | 1.00 (0.63-1.00) |  |
|  | 36-59 m | 0.39 (0.26-0.53) | 1.00 (0.78-1.00) | 0.15 (0.06-0.31) | 0.31 (0.19-0.46) | 1.00 (0.54-1.00) |  |

**Multiple measurement attempts:** Because each measurement was captured 3 times in a row, except annotated respiratory rate, agreements were also evaluated by measurement attempt, which are shared below.

**Overall percent agreement by measurement attempt (M1-3) and age category.**

|  | M | 0-1 month | 2-11 months | 12-35 months | 36-59 months |
| --- | --- | --- | --- | --- | --- |
| Hypoxemia |  |  |  |  |  |
| Device 1 | All | 0.91 (0.87 - 0.93) | 0.89 (0.85 - 0.92) | 0.97 (0.95 - 0.99) | |
|  | 1 | 0.88 (0.82 - 0.93) | 0.88 (0.82 - 0.93) | 0.97 (0.93 - 0.99) | |
|  | 2 | 0.91 (0.86 - 0.95) | 0.92 (0.86 - 0.96) | 0.95 (0.90 - 0.98) | |
|  | 3 | 0.92 (0.87 - 0.96) | 0.86 (0.79 - 0.92) | 0.99 (0.96 - 1.00) | |
| Device 2 | All | 0.78 (0.73 - 0.82) | 0.85 (0.81 - 0.88) | 0.96 (0.94 - 0.98) | |
|  | 1 | 0.72 (0.64 - 0.80) | 0.86 (0.79 - 0.91) | 0.96 (0.91 - 0.98) | |
|  | 2 | 0.79 (0.70 - 0.85) | 0.84 (0.77 - 0.89) | 0.96 (0.91 - 0.98) | |
|  | 3 | 0.83 (0.75 - 0.89) | 0.84 (0.77 - 0.90) | 0.96 (0.91 - 0.98) | |
| Device 5 | All | 0.78 (0.73 - 0.82) | 0.84 (0.80 - 0.88) | 0.94 (0.91 - 0.96) | |
|  | 1 | 0.76 (0.67 - 0.84) | 0.82 (0.75 - 0.89) | 0.92 (0.86 - 0.96) | |
|  | 2 | 0.77 (0.67 - 0.84) | 0.86 (0.79 - 0.92) | 0.95 (0.90 - 0.98) | |
|  | 3 | 0.80 (0.71 - 0.87) | 0.84 (0.75 - 0.90) | 0.94 (0.89 - 0.97) | |
| Device 3 | All | NA | NA | 0.92 (0.89 - 0.95) | |
|  | 1 | NA | NA | 0.92 (0.86 - 0.96) | |
|  | 2 | NA | NA | 0.92 (0.87 - 0.96) | |
|  | 3 | NA | NA | 0.93 (0.87 - 0.97) | |
| Device 4 | All | NA | NA | 0.88 (0.85 - 0.91) | |
|  | 1 | NA | NA | 0.90 (0.84 - 0.94) | |
|  | 2 | NA | NA | 0.87 (0.80 - 0.92) | |
|  | 3 | NA | NA | 0.88 (0.80 - 0.93) | |
| Tachycardia |  |  |  |  |  |
| Device 1 | All | 0.92 (0.89 - 0.95) | 0.92 (0.89 - 0.95) | 0.95 (0.93 - 0.97) | |
|  | 1 | 0.92 (0.86 - 0.96) | 0.91 (0.86 - 0.95) | 0.98 (0.94 - 1.00) | |
|  | 2 | 0.91 (0.86 - 0.95) | 0.92 (0.87 - 0.96) | 0.95 (0.90 - 0.98) | |
|  | 3 | 0.94 (0.88 - 0.97) | 0.93 (0.87 - 0.97) | 0.92 (0.86 - 0.96) | |
| Device 2 | All | 0.89 (0.86 - 0.92) | 0.95 (0.93 - 0.97) | 0.95 (0.93 - 0.97) | |
|  | 1 | 0.88 (0.81 - 0.93) | 0.93 (0.87 - 0.96) | 0.97 (0.92 - 0.99) | |
|  | 2 | 0.89 (0.82 - 0.94) | 0.97 (0.92 - 0.99) | 0.96 (0.91 - 0.98) | |
|  | 3 | 0.92 (0.85 - 0.96) | 0.97 (0.93 - 0.99) | 0.94 (0.88 - 0.97) | |
| Device 5 | All | 0.91 (0.88 - 0.94) | 0.96 (0.94 - 0.98) | 0.95 (0.93 - 0.97) | |
|  | 1 | 0.91 (0.84 - 0.96) | 0.94 (0.88 - 0.97) | 0.95 (0.90 - 0.98) | |
|  | 2 | 0.88 (0.80 - 0.93) | 0.96 (0.91 - 0.99) | 0.94 (0.89 - 0.97) | |
|  | 3 | 0.96 (0.90 - 0.99) | 0.99 (0.95 - 1.00) | 0.97 (0.93 - 0.99) | |
| Device 3 | All | NA | NA | 0.94 (0.91 - 0.96) | |
|  | 1 | NA | NA | 0.93 (0.88 - 0.97) | |
|  | 2 | NA | NA | 0.94 (0.89 - 0.98) | |
|  | 3 | NA | NA | 0.93 (0.87 - 0.97) | |
| Device 4 | All | NA | NA | 0.88 (0.84 - 0.91) | |
|  | 1 | NA | NA | 0.89 (0.83 - 0.94) | |
|  | 2 | NA | NA | 0.85 (0.78 - 0.90) | |
|  | 3 | NA | NA | 0.89 (0.82 - 0.94) | |
| Fever |  |  |  |  |  |
| Device 1 | All | 0.87 (0.83 - 0.90) | 0.90 (0.87 - 0.93) | 0.85 (0.82 - 0.89) | |
|  | 1 | 0.86 (0.79 - 0.91) | 0.90 (0.84 - 0.94) | 0.86 (0.80 - 0.91) | |
|  | 2 | 0.89 (0.83 - 0.94) | 0.91 (0.85 - 0.95) | 0.87 (0.80 - 0.92) | |
|  | 3 | 0.85 (0.77 - 0.90) | 0.89 (0.82 - 0.94) | 0.82 (0.75 - 0.88) | |
| Device 5 | All | 0.88 (0.84 - 0.91) | 0.88 (0.85 - 0.91) | 0.82 (0.78 - 0.85) | |
|  | 1 | 0.87 (0.80 - 0.92) | 0.88 (0.81 - 0.92) | 0.81 (0.75 - 0.87) | |
|  | 2 | 0.89 (0.83 - 0.94) | 0.91 (0.85 - 0.95) | 0.83 (0.76 - 0.88) | |
|  | 3 | 0.88 (0.80 - 0.93) | 0.87 (0.80 - 0.92) | 0.82 (0.75 - 0.88) | |
| Device 4 | All | NA | NA | 0.85 (0.82 - 0.89) | |
|  | 1 | NA | NA | 0.83 (0.76 - 0.89) | |
|  | 2 | NA | NA | 0.86 (0.79 - 0.91) | |
|  | 3 | NA | NA | 0.87 (0.80 - 0.92) | |
| Fast breathing (by annotation) |  |  |  |  |  |
| Device 1 | All | 0.54 (0.41 - 0.67) | 0.78 (0.63 - 0.88) | 0.88 (0.70 - 0.98) | 0.91 (0.79 - 0.97) |
| Device 2 | All | 0.44 (0.30 - 0.60) | 0.81 (0.70 - 0.89) | 0.89 (0.72 - 0.98) | 0.72 (0.58 - 0.83) |
| Device 5 | All | 0.54 (0.41 - 0.67) | 0.50 (0.37 - 0.63) | 0.40 (0.24 - 0.58) | 0.39 (0.26 - 0.53) |
| Device 3 | All | NA | NA | 0.88 (0.69 - 0.97) | 0.81 (0.69 - 0.90) |
| Device 4 | All | NA | NA | 0.89 (0.67 - 0.99) | 0.70 (0.57 - 0.81) |
| Fast breathing (by acoustic) |  |  |  |  |  |
| Device 1 | All | 0.77 (0.72 - 0.82) | 0.89 (0.84 - 0.92) | 0.88 (0.80 - 0.94) | 0.80 (0.74 - 0.85) |
|  | 1 | 0.81 (0.72 - 0.88) | 0.87 (0.78 - 0.92) | 0.92 (0.78 - 0.98) | 0.84 (0.74 - 0.91) |
|  | 2 | 0.75 (0.65 - 0.84) | 0.89 (0.81 - 0.94) | 0.84 (0.67 - 0.95) | 0.78 (0.67 - 0.87) |
|  | 3 | 0.74 (0.64 - 0.83) | 0.91 (0.82 - 0.96) | 0.88 (0.69 - 0.97) | 0.78 (0.69 - 0.87) |
| Device 2 | All | 0.81 (0.76 - 0.85) | 0.89 (0.85 - 0.92) | 0.85 (0.78 - 0.90) | 0.69 (0.62 - 0.75) |
|  | 1 | 0.81 (0.72 - 0.87) | 0.89 (0.82 - 0.94) | 0.78 (0.65 - 0.89) | 0.64 (0.51 - 0.75) |
|  | 2 | 0.81 (0.72 - 0.88) | 0.90 (0.84 - 0.95) | 0.86 (0.73 - 0.94) | 0.76 (0.64 - 0.85) |
|  | 3 | 0.82 (0.73 - 0.89) | 0.88 (0.81 - 0.93) | 0.90 (0.78 - 0.97) | 0.67 (0.55 - 0.78) |
| Device 5 | All | 0.42 (0.36 - 0.48) | 0.47 (0.41 - 0.53) | 0.37 (0.29 - 0.46) | 0.30 (0.24 - 0.36) |
|  | 1 | 0.41 (0.31 - 0.52) | 0.54 (0.44 - 0.64) | 0.34 (0.21 - 0.49) | 0.32 (0.22 - 0.44) |
|  | 2 | 0.40 (0.29 - 0.50) | 0.47 (0.37 - 0.57) | 0.40 (0.26 - 0.55) | 0.28 (0.18 - 0.39) |
|  | 3 | 0.45 (0.34 - 0.57) | 0.39 (0.29 - 0.50) | 0.38 (0.23 - 0.54) | 0.30 (0.20 - 0.41) |
| Device 3 | All | NA | NA | 0.85 (0.78 - 0.90) | 0.82 (0.77 - 0.87) |
|  | 1 | NA | NA | 0.85 (0.72 - 0.93) | 0.85 (0.76 - 0.92) |
|  | 2 | NA | NA | 0.82 (0.69 - 0.91) | 0.83 (0.74 - 0.91) |
|  | 3 | NA | NA | 0.88 (0.74 - 0.96) | 0.78 (0.66 - 0.87) |
| Device 4 | All | NA | NA | 0.92 (0.84 - 0.97) | 0.77 (0.71 - 0.82) |
|  | 1 | NA | NA | 0.93 (0.78 - 0.99) | 0.75 (0.64 - 0.84) |
|  | 2 | NA | NA | 0.90 (0.73 - 0.98) | 0.77 (0.66 - 0.86) |
|  | 3 | NA | NA | 0.93 (0.76 - 0.99) | 0.80 (0.69 - 0.88) |
