# Supplementary figures and images for: Multimodal pulse oximeters to support the integrated management of childhood illnesses: A usability and diagnostic accuracy assessment from a multi-country hybrid type 2 study
Source: PLOS Glob Public Health. 2026 Mar 26;6(3):e0004655. doi: 10.1371/journal.pgph.0004655 (PMC13020799; doi:10.1371/journal.pgph.0004655)

**S1 Fig. Detailed usability results by device.**

Device 1:


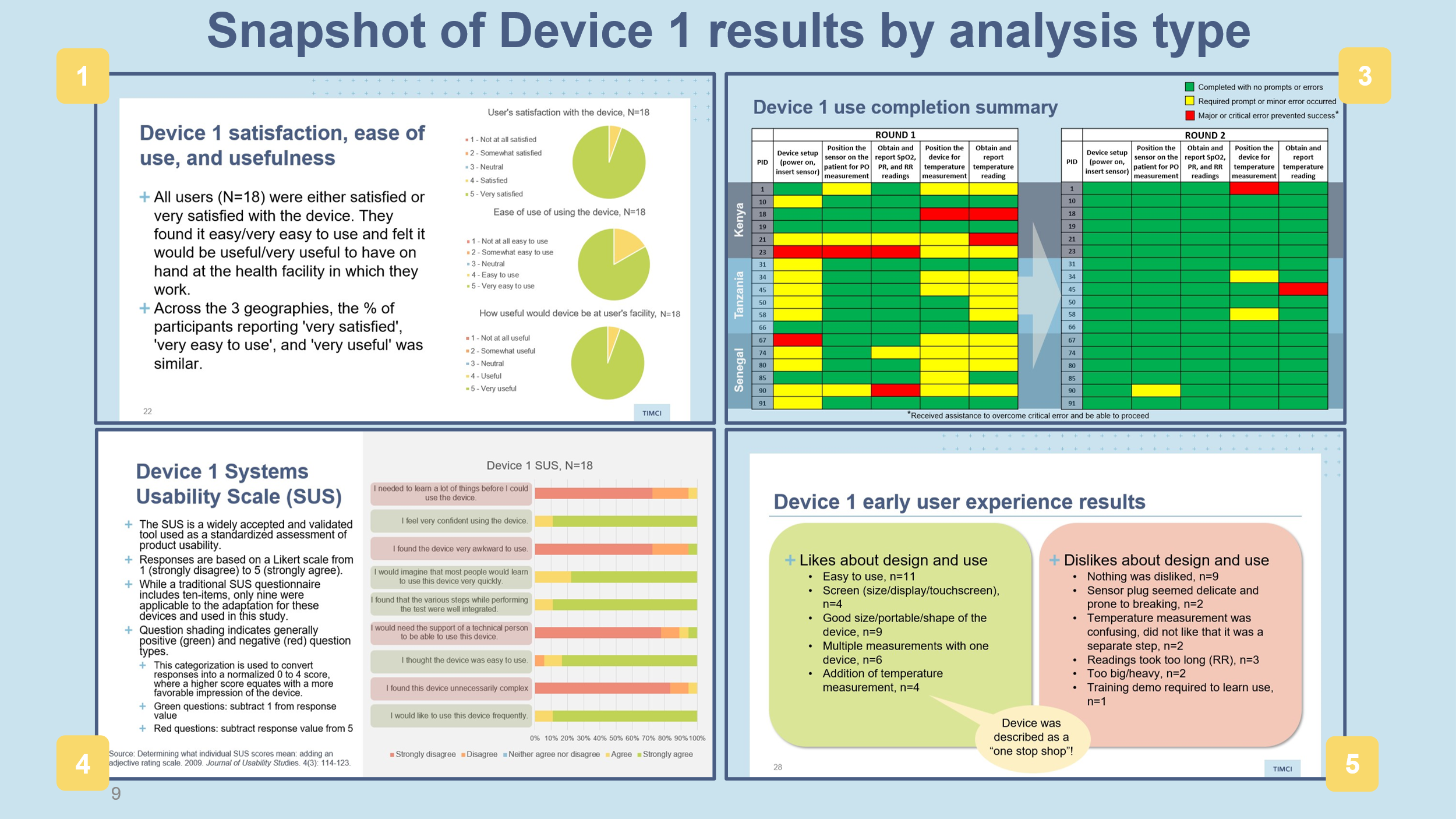


Device 2:


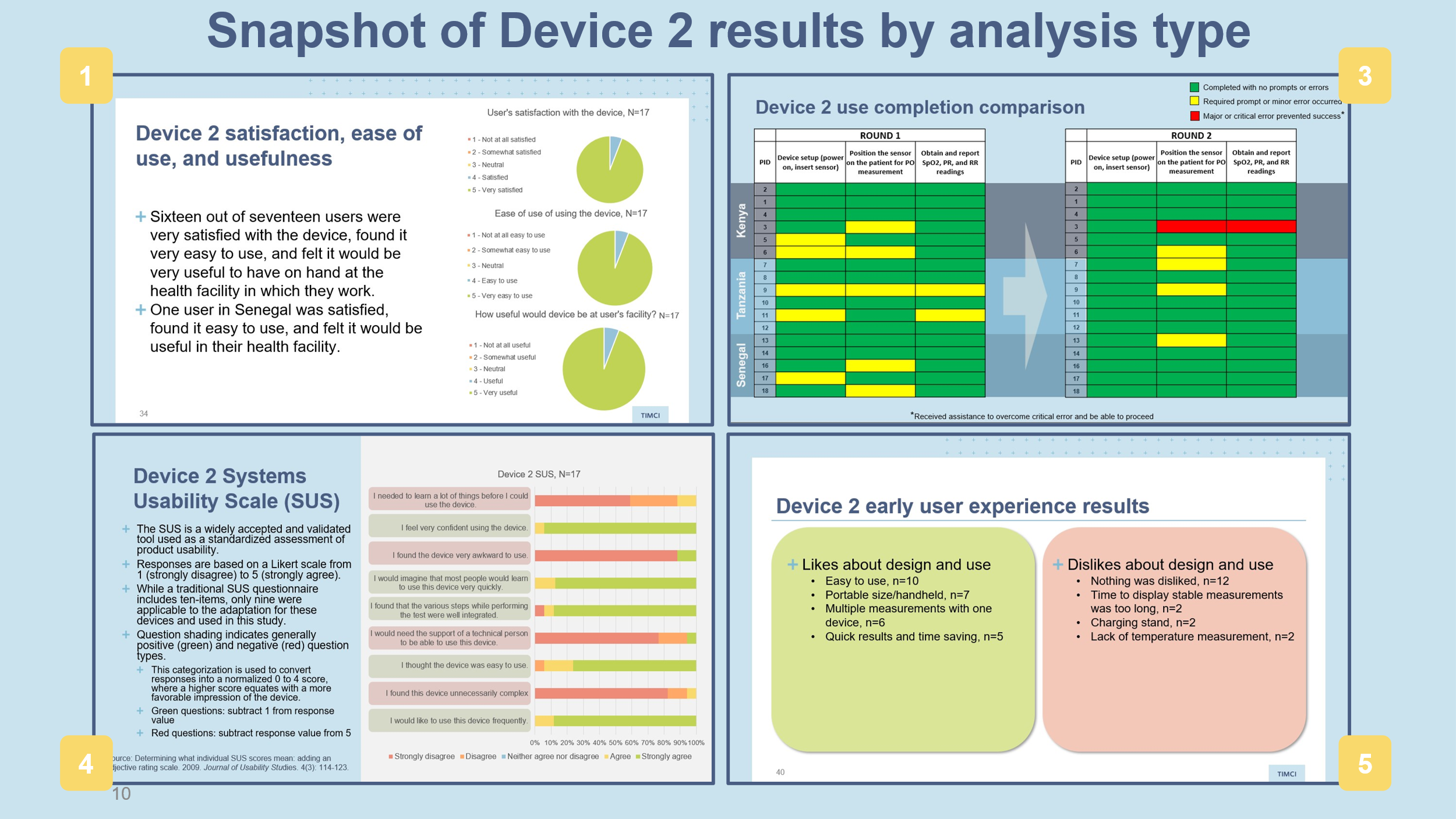


Device 3:


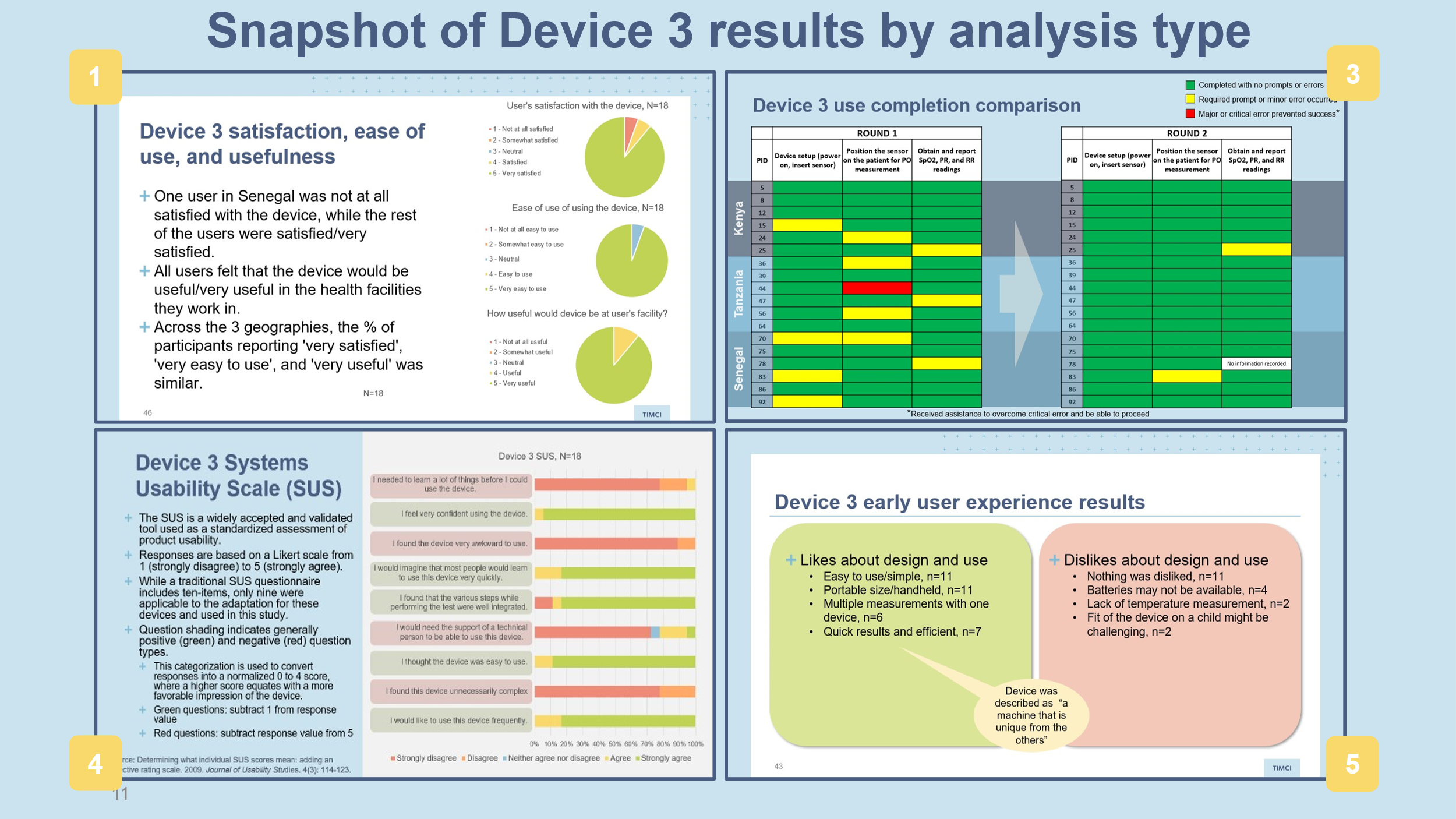


Device 4:


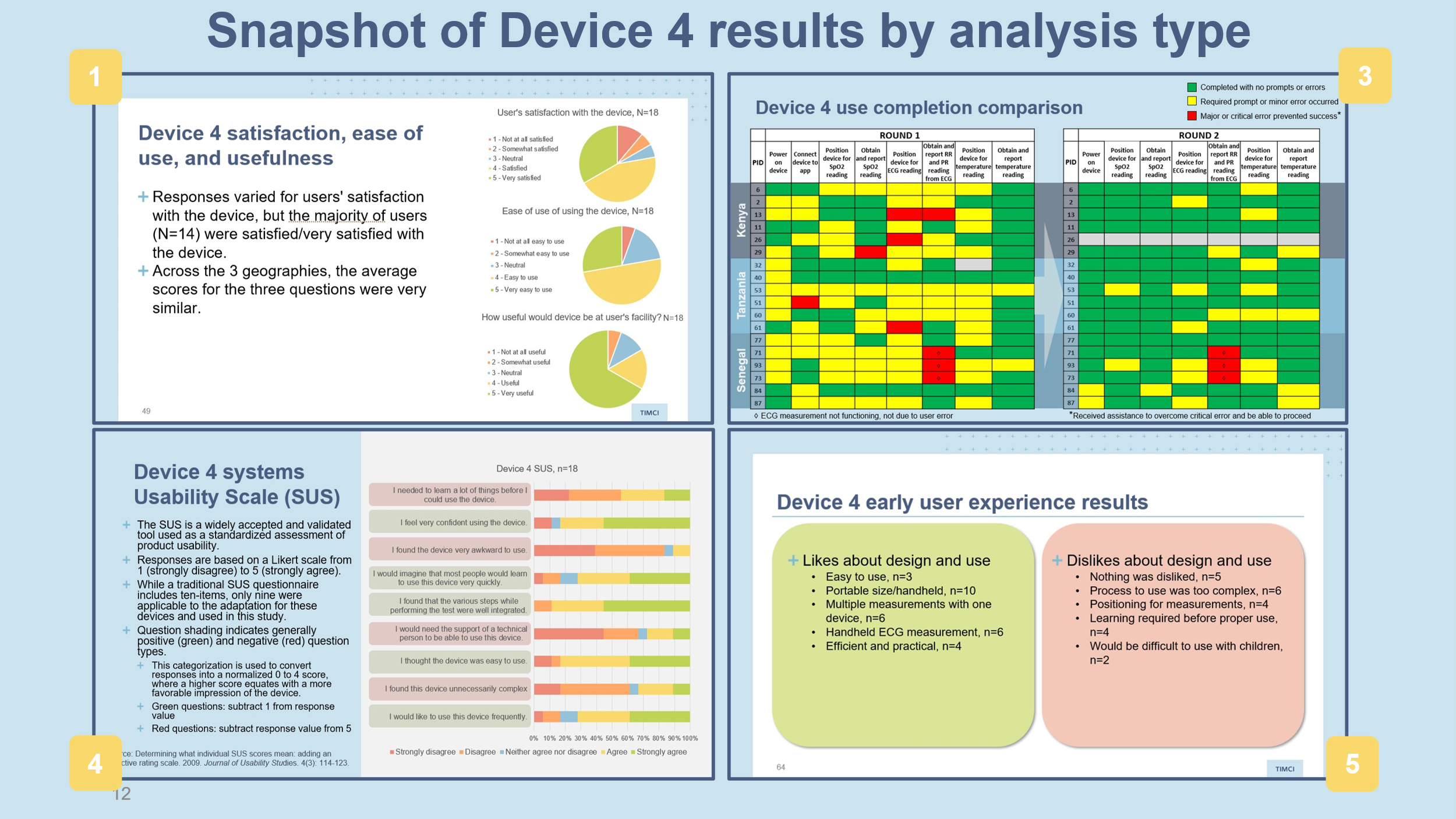


Device 5:
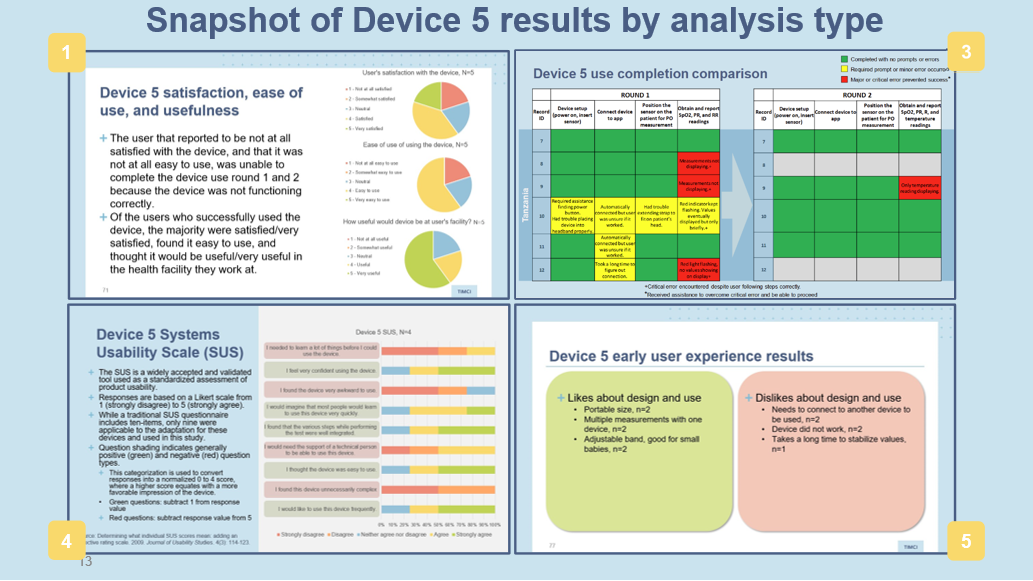


Device 6:


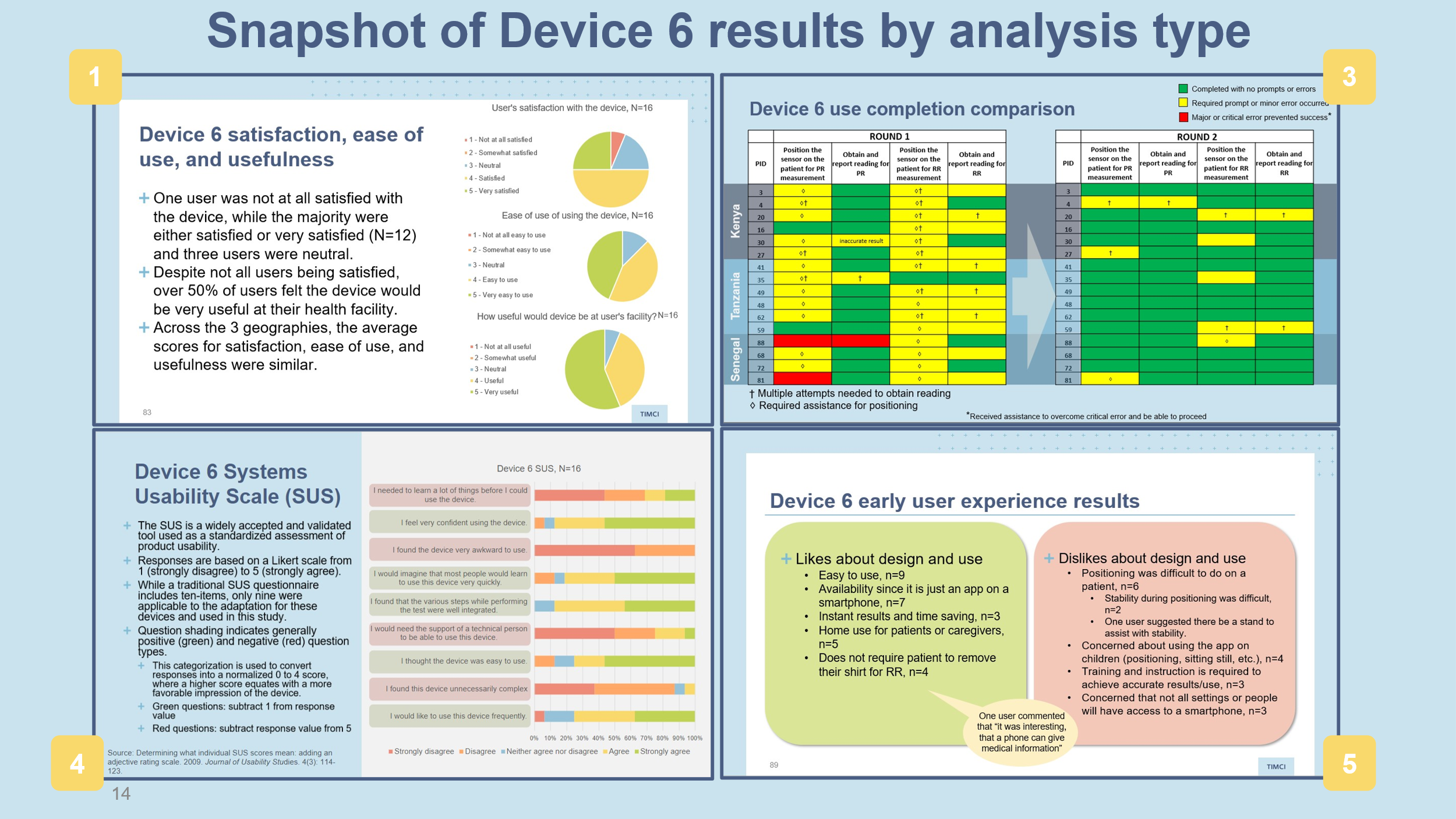

Supplement: S1 Fig — (DOCX) [file pgph.0004655.s006.docx]
